# A Brief History of Research on Mitotic Mechanisms

**DOI:** 10.3390/biology5040055

**Published:** 2016-12-21

**Authors:** J. Richard McIntosh, Thomas Hays

**Affiliations:** 1 Department of Molecular, Cellular and Developmental Biology, University of Colorado, Boulder, CO 80309, USA; 2 Department of Genetics, Cell Biology and Development, Medical School and College of Biological Sciences, University of Minnesota, Saint Paul, MN 55455, USA; haysx001@umn.edu

**Keywords:** mitosis, mitotic spindle, chromosome, kinetochore, microtubule, motor enzyme, centrosome, tubulin dynamics, force, accuracy

## Abstract

This chapter describes in summary form some of the most important research on chromosome segregation, from the discovery and naming of mitosis in the nineteenth century until around 1990. It gives both historical and scientific background for the nine chapters that follow, each of which provides an up-to-date review of a specific aspect of mitotic mechanism. Here, we trace the fruits of each new technology that allowed a deeper understanding of mitosis and its underlying mechanisms. We describe how light microscopy, including phase, polarization, and fluorescence optics, provided descriptive information about mitotic events and also enabled important experimentation on mitotic functions, such as the dynamics of spindle fibers and the forces generated for chromosome movement. We describe studies by electron microscopy, including quantitative work with serial section reconstructions. We review early results from spindle biochemistry and genetics, coupled to molecular biology, as these methods allowed scholars to identify key molecular components of mitotic mechanisms. We also review hypotheses about mitotic mechanisms whose testing led to a deeper understanding of this fundamental biological event. Our goal is to provide modern scientists with an appreciation of the work that has laid the foundations for their current work and interests.

## 1. Discoveries about Mitosis from Early Descriptions of Mitotic Structures

The history of research on mitosis is intertwined with the development of the relevant technologies, particularly microscopy. This linkage derives from the sizes of spindles and their activities; it also reflects a need for significant signal amplification to study mitotic components and processes. Moreover, the isolation of dividing nuclei as a simplified system for biochemical studies has proven technically difficult, so in the early days of mitosis research the majority of information came from work on whole cells. Indeed, research on mitosis has motivated the development of several microscope technologies, including more effective modes of live cell imaging. We have therefore organized this presentation around the emergence of relevant technologies and the physical sciences that enabled them.

**Figure 1:**
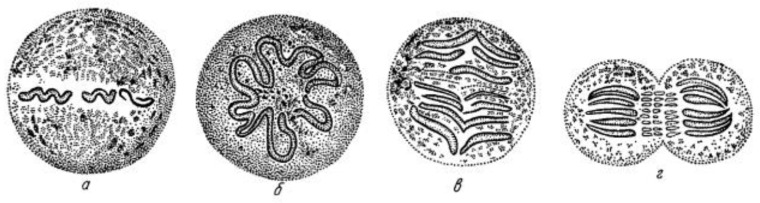
Drawing of dividing nuclei by Schneider, 1873 [1].

**Figure 2:**
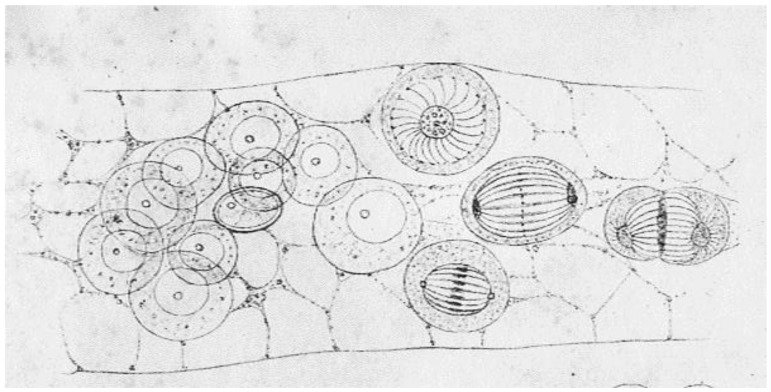
Drawing of mitotic figures that indicate structures at the spindle poles. van Benedin, 1876 [3] Image courtesy of Biodiversity Heritage Library. http://www.biodiversitylibrary.org. Drawing of mitotic figures that indicate structures at the spindle poles. van Benedin, 1876.

**Figure 3:**
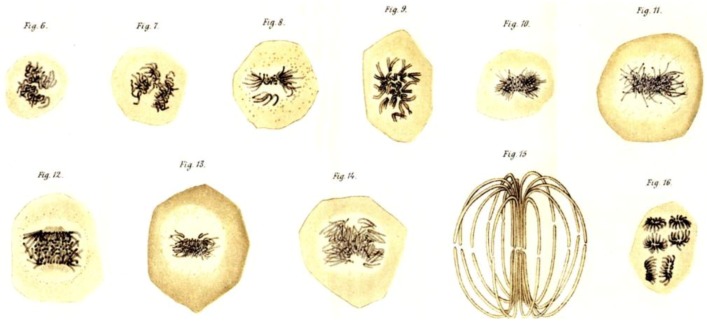
Drawings of mitotic figures by Flemming, 1878 [4,5]. This image is displayed under the terms of a Creative Commons License (Attribution-Noncommercial-Share Alike 3.0 Unported license, as described at http://creativecommons.org/licenses/by-nc-sa/3.0/.

Initial work on mitosis took place in several laboratories, beginning around 1870. Pioneering studies by Friedrich Schneider [1] (Figure 1), Eduard Strasburger [2] and others independently described the structures and positions of chromosomes in fixed, dividing cells, while Eduard Van Beneden [3] identified objects at the spindle poles that we would now call centrosomes (Figure 2). It was, however, Walther Flemming [4] (translated into English and republished [5]) who named the “mitotic” process and first described a plausible chronology of chromosome behavior in anticipation of cell division (Figure 3). Much of his work is assembled in an elegant book, published in 1882 [6].

Such work was possible because the imaging capabilities of the compound microscopes available at that time greatly exceeded those of the microscopes with which Hooke [7] first described cells in the 1660s, and with which van Leeuwenhoek [8] characterized the structures and behaviors of many single-celled organisms. Indeed, it was the invention of achromatic lenses (1823) that brought the resolution of light microscopy to ~1 µm, producing instruments that empowered Schleiden, a botanist, and Schwann, a zoologist, to demonstrate the ubiquity of cells, and Virchow [9] to realize that “all cells come from cells” (a powerful and important statement, despite its limited evolutionary perspective). They also provided both Pasteur and Koch with the tools they needed to recognize the importance of microorganisms in the propagation and progression of disease. Abbe’s invention of a high numerical aperture condenser in 1875 [10] and the subsequent introduction of oil immersion lenses (1878) finally brought microscopes to a space resolution of ~0.2 µm, enabling the remarkably accurate drawings found in the best of the early descriptions of mitosis. 

**Figure 4:**
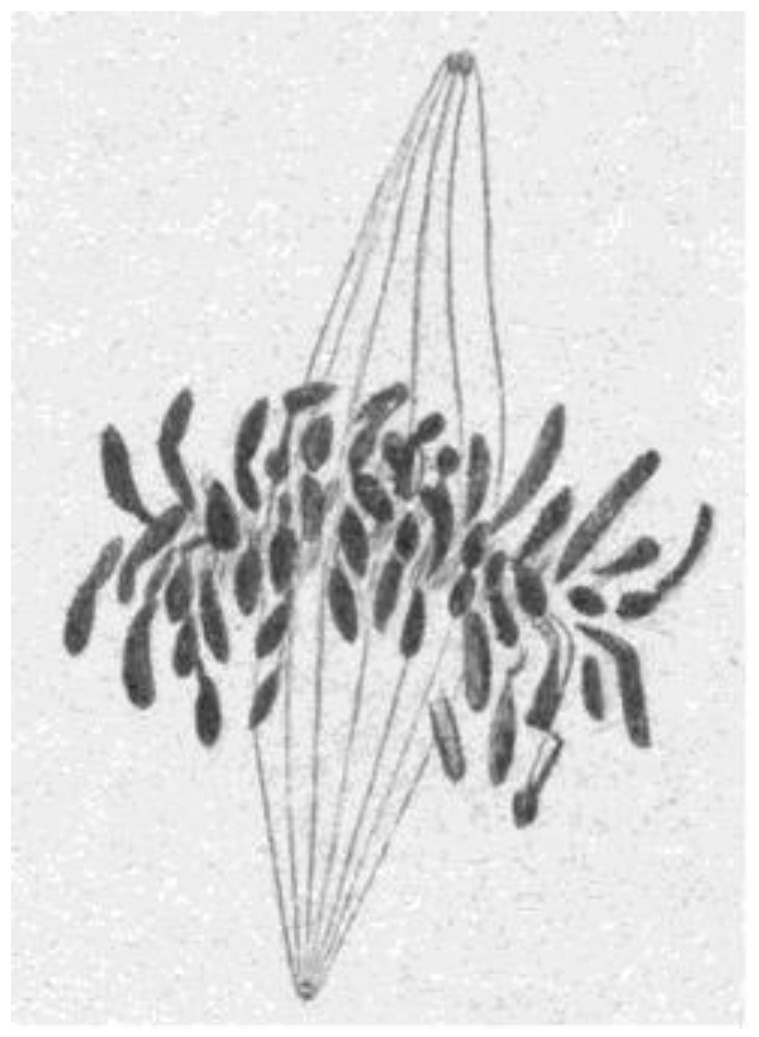
Drawing of a mitotic figure in a live cell prepared by Mayzel and published by Flemming, 1878. [4] This image is displayed under the terms of a Creative Commons License (Attribution-Noncommercial-Share Alike 3.0 Unported license, as described at http://creativecommons.org/licenses/by-nc-sa/3.0/.

**Figure 5:**
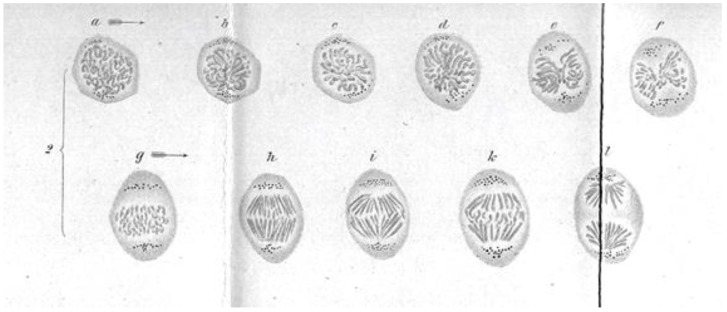
Drawings of chromosome segregation in living epidermal cells of a salamander larva. Flemming, 1878 [4]. This image is displayed under the terms of a Creative Commons License (Attribution-Noncommercial-Share Alike 3.0 Unported license, as described at http://creativecommons.org/licenses/by-nc-sa/3.0/.

These early studies explored a range of mitotic cells in tissues of both animals and plants, mostly in specimens that were fixed and stained prior to examination. In this situation, what one saw depended quite strongly on the method of sample preparation. Moreover, without a camera to record the observations, structures were represented by hand drawings. These factors, and the variations in structure across the range of specimens examined, led to disagreement about the validity of any given set of observations. The first verbal description of mitosis in living cells was given by Mayzel in 1875 [11]. With Mayzel’s permission, Flemming published a drawing of such a cell division [4] (Figure 4). Several other workers, such as Schleicher and Peremeschko, published images of chromosomes in live cells, but again it was Flemming who drew multiple stages in the division of a single cell type: epidermal cells from a salamander larva. The result was virtually a hand-drawn, time-laps movie (Figure 5) [4]. With time, additional images of mitosis in living cells were presented by scholars studying a range of organisms, and yet these descriptions generally involved only the chromosomes. At this point, there was no knowledge about the relationship between the “thick” fibers (chromosomes) and the “thin” ones (the spindle) seen in fixed material; both were thought to be manifestations of nuclear structure as this organelle prepared to divide.

The role of chromosomes as sites for the storage of a cell’s genetic information was first proposed by Weismann in 1885 [12]; in 1903–1904 Boveri [13] and Sutton [14] published studies on the behavior of chromosomes during both normal cell division and the generation of gametes. They realized independently that chromosome motions and patterns of segregation were consistent with the then controversial idea that chromosomes carried a cell’s genetic information. Most of these studies are well reviewed and summarized in the 1925 edition of E.B. Wilson’s monograph on “The Cell in Development and Heredity” [15], an important resource for students of mitosis. From all this descriptive groundwork, the essential features of chromosome segregation were established, but the underlying mechanism for mitosis was still mysterious. 

One limitation in this early work was the impact of the fixatives and stains used to visualize cellular infrastructure. Different fixation solutions were used by different investigators, but all such mixtures employed acids of various strengths and organic solvents, such as alcohols. Spindle fibers that might push and pull on chromosomes were seen by many, but only in fixed material, raising controversy about the legitimacy of these structures. While microscopists also saw mitotic events in living cells, in these specimens only the chromosomes were apparent. The very lack of visible spindle fibers in living cells cast doubt on the validity of the fibers seen in fixed cells, particularly since fixatives were known to induce the formation of aster-like structures in egg white and solutions of gelatin [16,17]. This observation led to the alternative concept that cytoplasm in living cells was colloidal, comprised of invisible particles and/or vesicles. In this view, fibers were artifacts of exposure to chemical fixatives, which triggered the condensation of invisible particles and/or vesicles into fibrous structures (reviewed in Wilson) [15].

The case for the reality of spindle fibers was supported by mitotic fibers that were evident in certain live cells, including diatoms as seen by Lauterborn in 1896 [18] (For an English translation, see [19]). Somewhat later, the case was enhanced by mechanical experiments in which Chambers used a microneedle to probe intracellular structures [20] (reviewed in [15]). These micromanipulation experiments showed that a spindle behaved as a coherent structure when twisted, rotated, displaced, or moved. The invention of phase optics by Zernike in the early 1930s (reviewed [21]), made spindle fibers more readily visible in some living cells, e.g., the flagellates living in the hind gut of the wood-feeding roach, *Cryptocercus* [22]. This work was particularly valuable, because the centrosomes in these unicellular organisms were much bigger than in most cells, allowing the first characterization of centrosome duplication and segregation during the cell cycle. Many workers in the field, however, viewed these results from “unusual cells” as unconvincing anomalies. Where were the spindle fibers in the mitotic cells of sea urchins, nematodes, amphibians, and mammals that had been the focus of so many studies?

**Figure 6:**
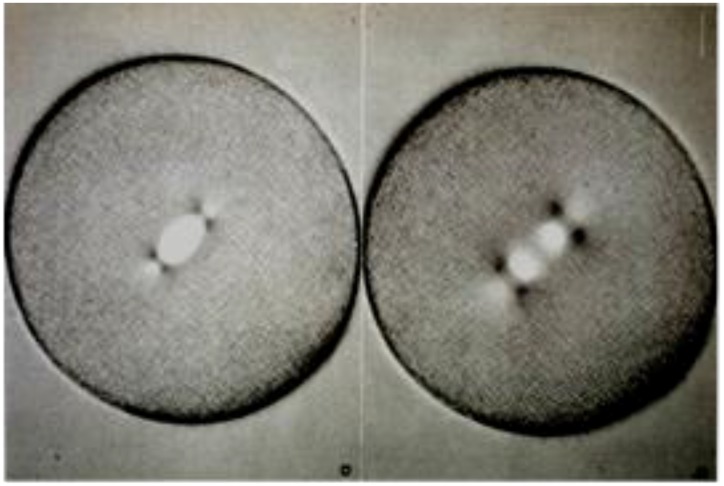
Mitotic spindles in living sea urchin eggs: metaphase (**left**) and mid-anaphase (**right**) viewed with polarization microscopy, similar to Inoue and Dan, 1951 [26]. Image from Salmon, E.D., 1982, Meth. Cell Biol. 25: 69–105. With permission from the author and the Copyright Clearance Center.

Another imaging breakthrough came from the work of W.J. Schmidt [23] and F.O. Schmidt [24], each of whom employed polarized light microscopy to visualize the birefringence (BR), i.e., the two refractive indices that are visible in optically anisotropic materials. Viewed between crossed polarizers, the apparently homogenous material surrounding mitotic chromosomes was clearly if weakly birefringent, evidence for the presence of fibrous material in the living spindle. Spindle BR was also seen in mitotic cells from vertebrates in 1948 by Hughes and Swann [25]. Shinya Inoue pioneered several advances in the optics used to detect and measure BR, enhancing the value of polarized light microscopy for detailed observations on mitosis in living cells. He invented a way to compensate for the position-dependent optical activity of high numerical aperture lenses, allowing him to visualize spindle BR with high sensitivity (which depends on the extinction of the polarizing system) and at comparatively high space resolution (which depends on numerical aperture) (Figure 6) [26,27]. This invention also allowed Inoue to experiment with the factors that increased and decreased the amount of spindle BR [26], as described in more detail below. With this technology, Inoue saw time-dependent fluctuations in spindle birefringence and was able to use cinematography to capture the entire process of mitosis in livings cells from both plants and animals. These innovations led to an essentially universal acceptance of spindle fibers as a reality. 

## 2. New Technologies for Structural Studies Advanced Our Understanding of Spindle Organization

**Figure 7:**
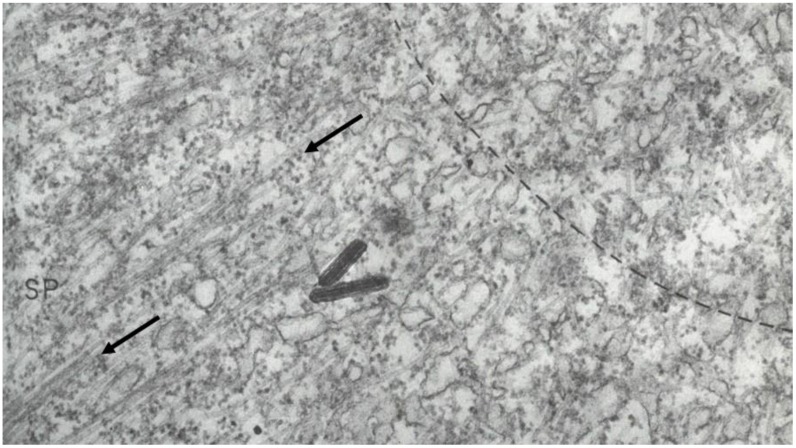
A portion of a sea urchin mitotic spindle (SP) imaged in an electron microscope, showing the MTs (arrows) that make up the spindle fibers that had been seen by light microscopy. The curved dashed line marks the polar end of the spindle and the beginning of a specialized region that surrounds the spindle pole in these cells. (Dark rods are contamination.) Harris, 1965 [30]. This image is displayed under the terms of a Creative Commons License (Attribution-Noncommercial-Share Alike 3.0 Unported license, as described at http://creativecommons.org/licenses/by-nc-sa/3.0/.

**Figure 8:**
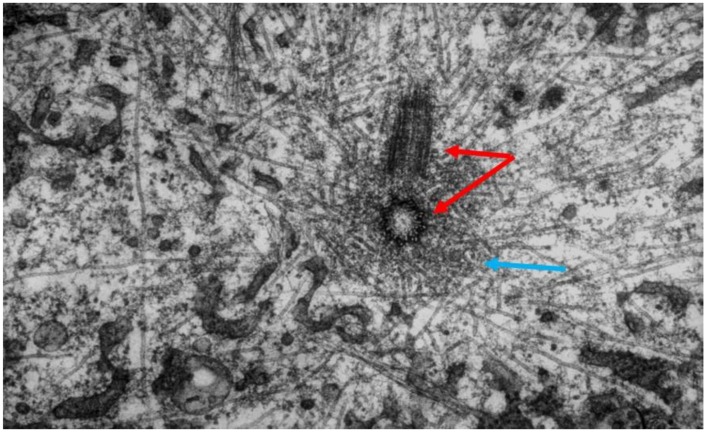
Electron micrograph of a mitotic spindle pole in a cultured mammalian cell, fixed with glutaraldehyde. Red arrows mark centrioles, the blue arrow indicates pericentriolar material where MTs are nucleated. Image kindness of Kent McDonald, Univ. California, Berkeley.

**Figure 9:**
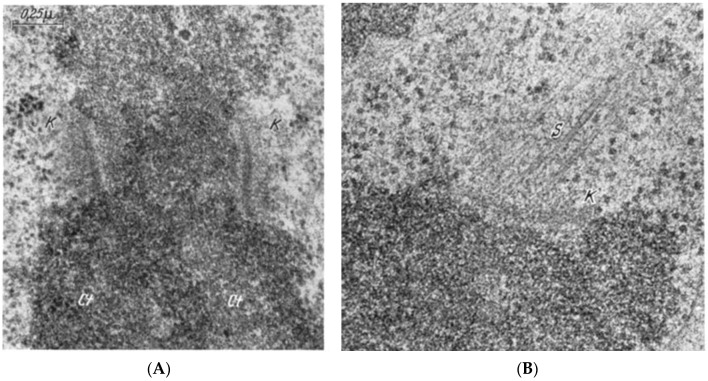
Kinetochores (*K*) are the specializations on mitotic chromosomes (*Ct*) that bind MTs. (**A**) = sister kinetochores in a mammalian cell, strain CHO, treated with colcemid to block MT formation; (**B**) = a kinetochore after removal of the drug and regrowth of spindle MTs (*S*). From Brinkley and Stubblefield, 1966 [33]. With permission from Elsevier Publishing.

The visualization of spindle fibers took another step forward when mitotic cells were successfully studied by electron microscopy. Initial work used the same harsh fixations that had produced fibers for view in the light microscope; now seen at higher resolution, the fibers appeared as bundles of much finer fibrils [28,29]. The “fine structure” of these fibrils was later seen with greater clarity by Harris in sea urchin blastomeres [30] (Figure 7) and by Roth and Daniels in amebae [31] that had been fixed with osmium tetroxide, either at low pH or in the presence of divalent cations. In this work, spindle fibers corresponded to bundles of 15 nm filaments that appeared tubular. With the subsequent discovery of glutaraldehyde as a fixative [32], similar and better-preserved tubular fibers, now 25 nm in diameter, were found in all spindles studied (Figure 8). Some of these spindle “microtubules” (MTs) were seen by Brinkley and Stubblefield to attach to specializations on each chromatid of a metaphase chromosome. These specializations appeared as paired structures at the chromosome’s primary constriction or “centromere” (Figure 9) [33]. The attachment sites were identified as loci of MT binding and called “kinetochores”, a term given earlier to the chromosomal regions responsible for chromosome motion. The spindle thereby became visible as an organized assembly of MTs that must somehow exert forces on chromosomes. This idea has served ever since as the framework for most work on mitosis ever since. 

**Figure 10:**
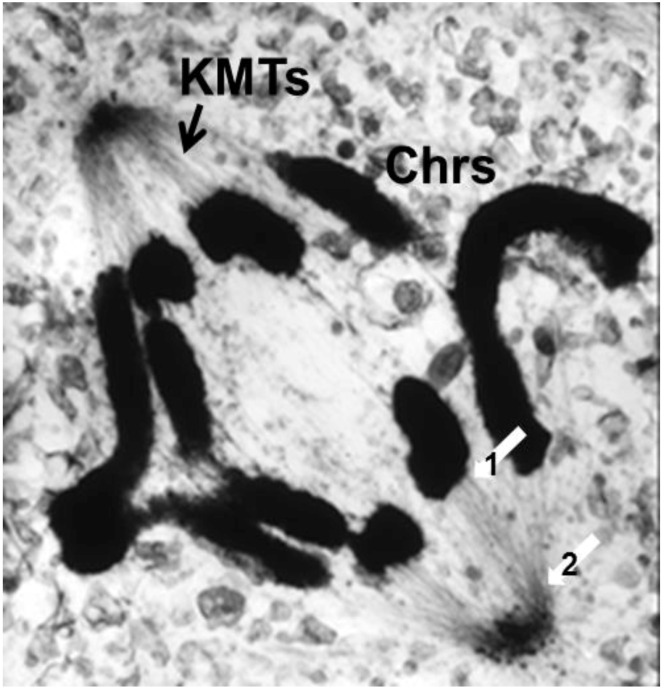
Thick section of a mammalian cell in anaphase, lysed before fixation to reduce the complexity of background staining. KMTs = kinetochore microtubules; Chrs = chromosomes. White arrows indicate sites of apparent attachment between MTs and a chromosome (1) and a pole (2). From McIntosh et al., 1975b [37]. By permission of the author.

**Figure 11:**
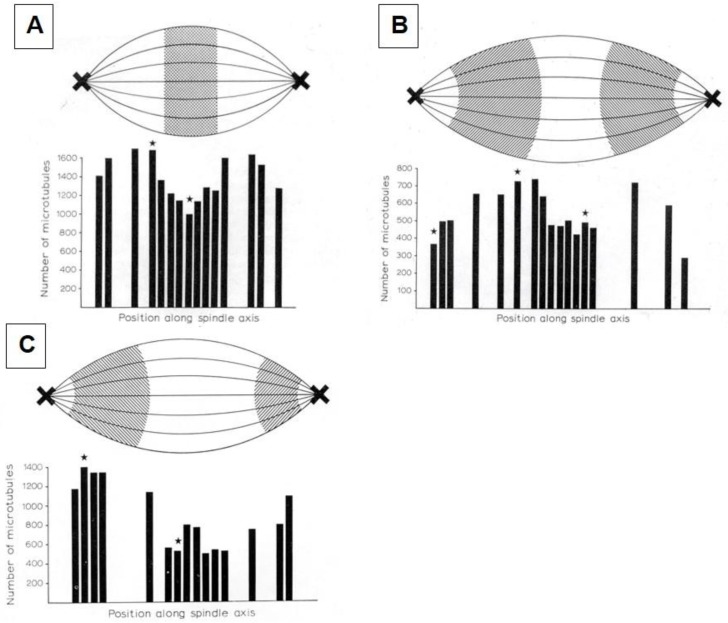
Counts of total numbers of MTs seen on successive spindle cross-sections from pole to pole at three stages of mitosis: (**A**) = metaphase; (**B**) = early anaphase; (**C**) = mid anaphase. From McIntosh and Landis, 1971 [34]. This image is displayed under the terms of a Creative Commons License (Attribution-Noncommercial-Share Alike 3.0 Unported license, as described at http://creativecommons.org/licenses/by-nc-sa/3.0/.

**Figure 12:**
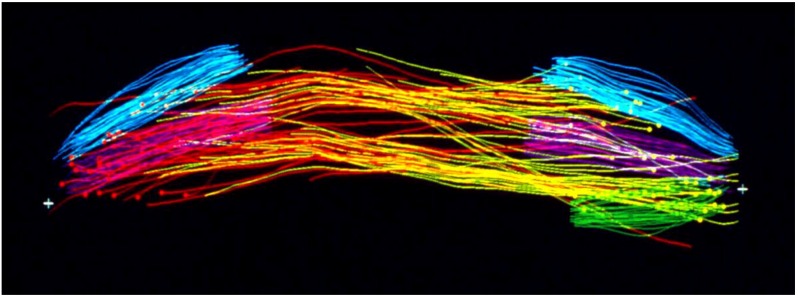
The paths of many spindle MTs, traced through serial sections of a mammalian cell (strain PtK2) in anaphase. White crosses mark the positions of the poindle poles. The shorter bundles of colored lines represent the kinetochore MTs that cluster to form the kinetochore fibers visible in the light microscope. (Colors are used simply to make these clusters stand out.) The red and yellow lines represent non-kinetochore MTs, which are associated with one pole or the other and interdigitate at the spindle’s midplane to make the “interpolar” spindle. These MTs slide and elongate during anaphase B. Image kindness of D. Mastronarde, Univ. Colorado.

Considerable effort has gone into the structural characterization of the spindle’s MT component. Most of the early work used electron microscopy of serial sections cut from fixed and plastic-embedded samples; this approach provided the resolution in 3-dimensions (3-D) necessary to distinguish the individual but tightly bunched MTs and to reveal the overall architecture of spindle fibers (Figure 10). Initial quantitative work on spindle structure was based on counts of the numbers of MTs in spindle cross-sections, presented as a function of position along the spindle axis, which was assessed by the number of sections cut since the one that included a spindle pole (Figure 11A–C) [34,35,36]. As techniques improved, investigators were able to track each MT from section to section, allowing displays of some aspects of spindle geometry in 3-D, e.g., the interdigitation of MTs associated with each half of the spindle in the region near the midplane of an anaphase spindle. This arrangement formed a robust interpolar bundle, the structure seen by light microscopy as the “continuous” or “pole-to-pole” spindle [34,37] (Figure 12). 

Some students of spindle structure used thin sections cut parallel to the axis of the spindle and traced each MT as it appeared on a single section; they then super-imposed these traces to make a representation of spindle structure that served useful comparative purposes. Although these views were drawings, not full reconstructions of spindle organization in a sub-volume of the overall structure, they provided informative views of the spindle after an experimental treatment [38]. 

**Figure 13:**
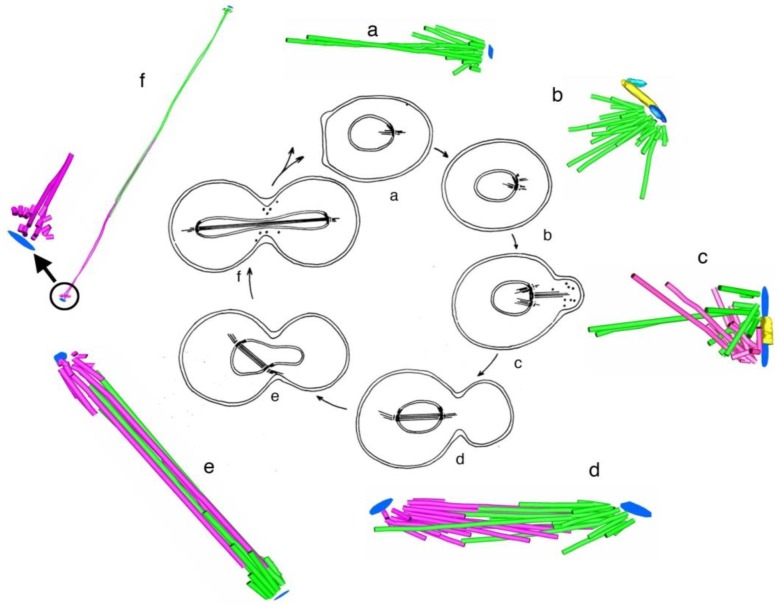
The spindle cycle in budding yeast. In the center of the figure, drawings represent the structure of budding yeast cells as they traverse the cell cycle. Around the edges are models made from tomographic reconstructions of the MT component of yeast spindles at each stage of mitosis. (**a**) There is only one centrosome but MTs grow from it into the cell’s nucleus; (**b**) The centrosome is duplicated and more shorter MTs project into the nucleus; (**c**) There are now two functional centrosomes, sitting side by side, each projecting MTs into the nucleus. At this stage, the spindle is in the process of attaching sister kinetochores to sister spindle poles; (**d**) A bi-polar spindle has formed; (**e**) The cell is advanced in anaphase B and the sister chromosomes are well separated; (**f**) A long, slender spindle runs from pole to pole (green and magenta MTs), and the chromosomes are drawn tightly around each pole. This spindle severs as the cell divides at cytokinesis, and the cell returns to state A. Redrawn from [43] by Eileen O’Toole. This image is displayed under the terms of a Creative Commons License (Attribution-Noncommercial-Share Alike 3.0 Unported license, as described at http://creativecommons.org/licenses/by-nc-sa/3.0/.

The small spindles found in micro-organisms provided a particularly fruitful field for study by electron microscopy. The first group to capitalize on these cells used high voltage electrons to image comparatively thick sections cut parallel to the spindle axes in cells that had been lysed during fixation, removing much of the cytoplasmic density that is characteristic of small cells [39]. With stereo views at distinct stages of spindle formation and function, one could get a good overview of spindle organization and its changes with time as the MTs grew from the centrosomes, formed a bi-polar array, then organized the chromosomes and segregated them, largely through spindle elongation. A more detailed view of spindles in small cells emerged from the use of larger numbers of serial thin sections cut perpendicular to the spindle axis. With these sections one could track MTs in 3-D to characterize their distribution. The well-ordered interpolar spindles of diatoms were the first to yield information about changes in MT arrangement as a function of spindle elongation in anaphase B [40]. Subsequent work extended these discoveries to other diatoms, then to a cellular slime mold [41] and budding yeast [42]. This work, in sum, revealed a consistent pattern of structure in which one or a few MTs associated end-on with each chromosome, and a bundle of MTs formed between the two spindle poles, setting up an interdigitating framework of anti-parallel MTs whose interactions near the spindle midplane could drive spindle elongation through the sliding apart of two MT families, commonly accompanied by MT growth (Figure 13).

An interesting reflection on the value of technological improvements in the progress of mitosis research is seen in a comparison of the results cited above with work done after the power of fluorescence microscopy became widely appreciated. Studies of budding yeast spindles by indirect immunofluorescence produced images quite like those that were laboriously prepared by serial section electron microscopy [43]. In the early work the resolving power of the electron microscope was used largely for fiber classification (MTs vs. microfilaments, etc.) and to resolve closely spaced fibers; such detail was not necessary for the study of gross fiber motions. Later chapters of this book will show how the clever use of light microscopy, together with various techniques for image contrast generation, have contributed tremendously to our current understanding of spindle mechanics.

**Figure 14:**
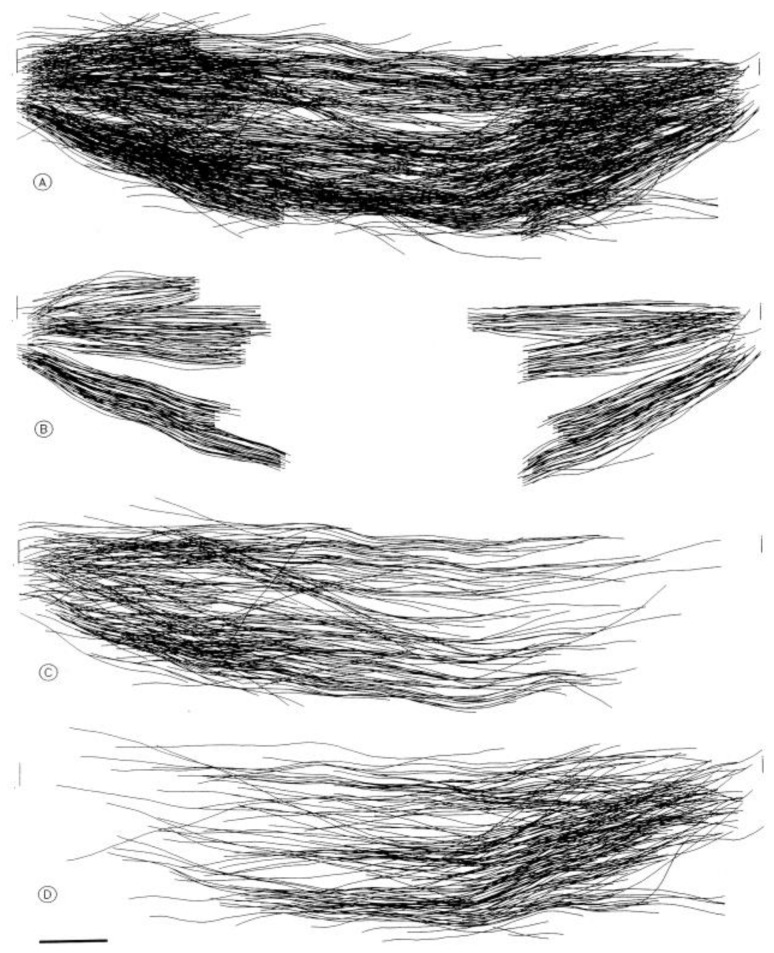
Two-dimensional projections of all the MTs in a volume that includes ~one-half of an early anaphase spindle from a PtK1 cell. (**A**) = all MTs traced; (**B**) = all kinetochore-associated MTs seen; (**C**,**D**) = all non-KMTs associated with the two spindle poles. From Mastronarde et al., 1993 [47]. This image is displayed under the terms of a Creative Commons License (Attribution-Noncommercial-Share Alike 3.0 Unported license, as described at http://creativecommons.org/licenses/by-nc-sa/3.0/.

Serial cross-sectioning and electron microscopy also provided important early information about the MT components of bigger spindles. The structure of the cold-stable bundle of MTs that associates with each kinetochore in a mammalian cells was elucidated in this way [44], and the structure of both kinetochore-associated MTs [45] and other spindle MTs that contribute to mammalian spindle structure [46] were similarly studied (Figure 14A–D). Unlike the small spindles, in which all MTs had one end on a spindle pole, these larger structures included many MTs, both of whose ends appeared free in the body of the spindle. Moreover, not all of the MTs with one end on a kinetochore were long enough to reach the area around the pole. In addition, the kinetochore-associated fibers visible in the light microscope were seen to contain MTs that did not end on a kinetochore. This structural complexity challenged the perspective that chromosome segregation was accomplished in all cells by a common mitotic mechanism. Instead, the structural variation suggested that spindles did not simply scale up in size; big spindles might involve different structural and functional principles than small ones. Perhaps bigger cells with more and bigger chromosomes placed different demands on spindle mechanics, so different solutions for chromosome segregation were required.

**Figure 15:**
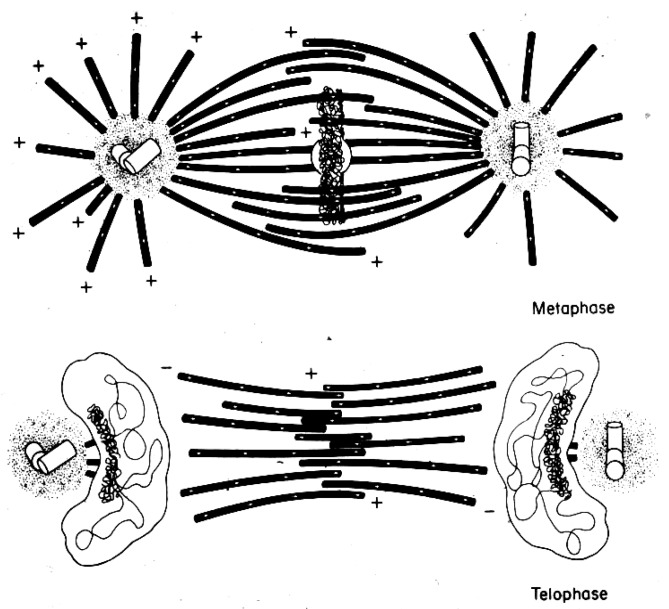
Diagrams showing the polar orientation of Spindle MTs, as assessed by the tubulin-containing hooks. Euteneuer and McIntosh 1981, 1982 [56,57]. This image is displayed under the terms of a Creative Commons License (Attribution-Noncommercial-Share Alike 3.0 Unported license, as described at http://creativecommons.org/licenses/by-nc-sa/3.0/.

An important limitation with all the early structural studies of spindle MTs was their inability to detect the polar orientation of these polymers. MTs were demonstrated to be polar, i.e., to be vectors, by Amos and Klug [47], although the concept of MT polarity had been identified as important for mitosis somewhat earlier [48]. There were indications from experiments, both in vivo [49,50] and in vitro [51,52], that MTs could grow from either the centrosomes at the spindle pole or the kinetochores of metaphase chromosomes, suggesting that the MTs in any half-spindle pointed in opposite directions. The issue of spindle MT polarity was settled in several steps: (1) Experiments in vitro revealed a kinetic polarity in MT growth; the polymers had a fast and a slow growing end [53]. Work from the Borisy lab showed that MTs growing from centrosomes were oriented with their fast-growing “plus” ends distal to the centrosome [54]; (2) A method was discovered by which the protein subunit of MTs would add to the walls of existing MTs, forming hooks whose direction of curvature revealed the polar orientation of the original MT lattice [55]; (3) The application of hooks to spindles showed that both the MTs emanating from the spindle poles and those associated with kinetochores were oriented in the same direction: their fast-growing ends were distal to the spindle pole [56,57]; (4) The flagellar ATPase, dynein was identified as an additional polarity marker, binding along the MT lattice in a polarized fashion and confirming the underlying MT orientation in spindles [58]. Thus, the polar orientation of spindle MTs turned out to be strikingly simple (Figure 15). The ability of kinetochores to promote the nucleation of MTs then posed a mystery: are these MTs initiated upside down or does the spindle contain some MTs that are oppositely oriented? Intriguingly, the structural evidence argued strongly against the latter possibility, but exactly how kinetochores can initiate MTs of the right orientation is still an unsolved problem.

## 3. Comparisons of Spindles across Phyla

The fact that mitosis occurs in all eukaryotic cells has meant that students of diverse organisms have contributed to the literature of the field. Although the earliest studies were focused on organisms whose cells were comparatively large and whose chromosomes were particularly visible, e.g., the amphibians, insects, nematodes, oocytes of marine invertebrates, and certain plants, subsequent investigations reached out more widely. Cells from the endosperm in plant seeds have been particular useful because they make almost no cell walls, which improves both the clarity of images obtained by light microscopy and the ease with which cells can be flattened to reduce their 3-D image complexity. One of the most productive scholars of plant mitosis was Andrew Bajer, who initially used phase microscope with cine-recording, then polarization optics, then electron microscopy (reviewed in [59]) and subsequently immune-staining of spindle components to characterize mitosis in these large and beautiful cells [60]. Bajer and his students described large bundles of MTs associated with each chromosome and many additional spindle MTs, assembled into a structure that resembled the interpolar spindle of animal cells, except for its lack of a focus on defined polar structures.

**Figure 16:**
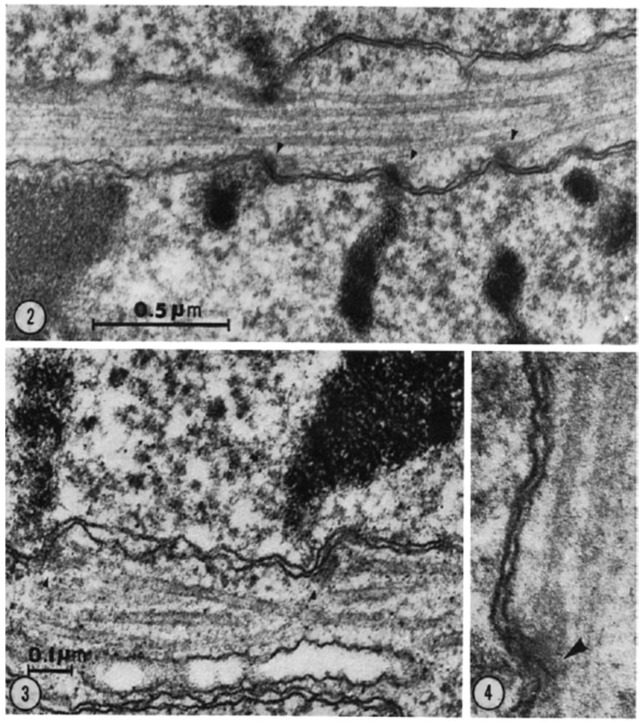
MTs, chromosomes and their interactions in the dinoflagellate, *Amphidium*. MTs run in a cytoplasmic channel, but some of them are connected to chromosomes through the nuclear envelope. From Oakley and Dodge [63]. This image is displayed under the terms of a Creative Commons License (Attribution-Noncommercial-Share Alike 3.0 Unported license, as described at http://creativecommons.org/licenses/by-nc-sa/3.0/.

Micro-organisms were also subjects for detailed and informative study. Hans Ris and Donna Kubai used electron microscopy to describe spindles in several dinoflagellates, which were thought at the time to represent a particularly primitive kind of eukaryote whose spindles might, therefore, give insight into the evolutionary origins of mitosis [61]. These organisms carried out mitosis within their nucleus, and the images then available suggested that cytoplasmic MTs were simply a guide for the direction that chromosomes might move. It was proposed that motive force might come from the membranes of the nuclear envelope, by analogy with a then popular model for the mechanism of chromosome segregation in bacteria [62]. In retrospect, the apparent absence of connections between chromosomes and MTs may have reflected procedural difficulties in preserving these cells for structural studies. This view is supported by the observation that in at least some dinoflagellates MTs terminate on a knob-like differentiation at the site of contact between kinetochores and the nuclear envelope [63] (Figure 16). Some of the difficulties of preparing micro-organisms for electron microscopy have been solved by the introduction of rapid freezing, followed by fixation through the substitution of cytoplasmic water with organic solvents at low temperature (freeze-substitution) [64]. In the study of cellular fine structure, these methods provided a significant improvement in cell preservation [65]. 

The biological diversity of micro-organisms has been informative about different ways in which organisms solve the problem of reliable chromosome segregation [66,67]. However, this knowledge has been a two-edged sword; it has provided a legitimate sense of variation but also a bewildering sense of complexity. The wide range of mitotic and meiotic structures and processes has nurtured the belief that there could not possibly be a “universal” mechanism for chromosome movement. For clarity in addressing this issue, a brief summary of mitotic and meiotic events is presented here, followed by some examples of diversity in the patterns of chromosome motion. 

## 4. A Summary Description of Mitotic and Meiotic Events

Despite diversity in the patterns of chromosome segregation, some common features have been identified. Small spindles, as found in most fungi and algae, build a distinct interpolar spindle (sometimes called the central spindle, the continuous spindle, or the core bundle, referring to its tightly bunched MTs). One or more MTs that are not in this bundle run directly from one of the spindle poles to each kinetochore, making a mechanical connection that is essential for normal chromosome motion. Commonly, the chromosomes congress to a metaphase plate, but the connection between sister chromatids is not rigid, allowing the metaphase chromosomes to separate transiently, showing a “breathing” as they move back and forth about the spindle equator [68]. In some diatoms, this breathing is so extreme that sister kinetochores are pulled to opposite spindle poles before anaphase begins [69]. The onset of anaphase is usually abrupt [70], and most chromosomes separate due to two kinds of motions: an approach to the spindle poles (Anaphase A) and an elongation of the distance between the poles (Anaphase B). 

In medium sized spindles, such as those in the cells of mammals, nematodes, and fruit flies, there are multiple MTs (5–40) attached to each kinetochore, and some but not all of them run from kinetochore to pole [71]. Again, there is an interpolar spindle, but not all of the MTs in this structure have one end associated with a pole; many of them start and end in the body of the spindle, and some of them commingle with the kinetochore-attached MTs, making the “kinetochore fiber” that is visible by light microscopy a mixture of MTs that are kinetochore associated and those that are not [46]. While centrosomes are present and contribute to spindle organization, they have been shown by several experiments to be dispensable, so long as slower mitotic progression can be tolerated. In large spindles, such as those that form in early frog embryos or in the endosperm of higher plants, there are many thousands of MTs. In frog eggs, most of these form in association with chromosomes, rather than centrosomes, and they form a bipolar spindle through a combination of MT rearrangements and dynamics, as will be discussed in later chapters of this book. Connections of MTs directly to the poles are rare. Thus, there appear to be several structures to understand more fully in our efforts to elucidate mitotic mechanisms. 

**Figure 17:**
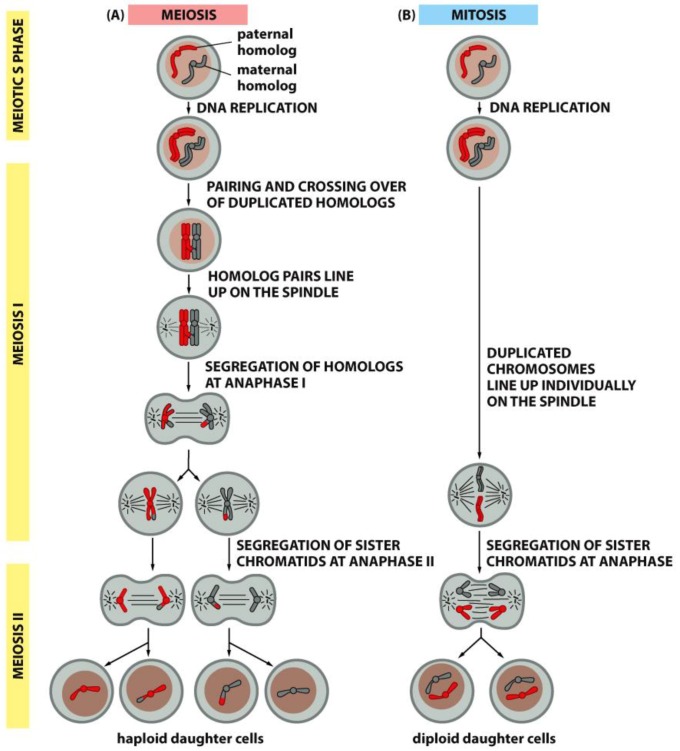
Diagrams of meiotic (**A**) and mitotic (**B**) cell divisions. With permission from the Taylor Francis Group, publishers.

Meiotic spindles resemble mitotic spindles in many ways, but the essential function of reducing the chromosome complement from diploid to haploid requires a few important differences. Foremost, there are two rounds of chromosome segregation with only one round of DNA replication. Secondly, in preparation for the first meiotic division, homologous chromosomes pair and become connected by the crossings-over that occur during meiotic prophase; a set of four chromatids (two identical and two homologous) form a single meiotic chromosome, called either a “bivalent” or a “tetrad” (Figure 17). These congress to a metaphase plate and segregate by the separation of “half-bivalents”, which serve as anaphase chromosomes for meiosis I. Following telophase, a short interphase that lacks DNA replication, the chromosomes enter meiosis II, which resembles a mitotic division, except that the number of chromosomes at the metaphase plate is half the number found in a diploid mitosis (Compare Figure 17, left and right).

Diversity in the mechanisms of chromosome segregation is found in several published works. For example, the first meiotic division of spermatocytes in crane flies includes a normal anaphase separation of paired autosomal bivalents, but a delayed separation of the two unpaired sex chromosomes [72]. The replicated but unpaired sex chromosomes, exhibit amphitelic attachments (i.e., sister kinetochores are attached to sister spindle poles). After autosomal segregation is complete, the sex chromosomes segregate almost simultaneously to opposite poles, retaining their amphitelic attachments as they move in opposite directions by a mechanism that remains unexplained. 

Another anomaly is found in the fly, *Sciara copraphilia*, where meiosis I in males includes segregation of the male-contributed chromosomes from the female chromosomes on a monopolar spindle, which discards the former and draws in the latter to a single spindle pole [73,74]. Again, the mechanism underlying this chromosome behavior is still mysterious. At various times, students of mitosis have focused on these unusual behaviors and demanded that any successful hypothesis for mitotic mechanism must explain all these unusual phenomena. With the advent of molecular biology and a subsequent focus on chromosome segregation in a relatively few “model organisms”, this view has faded. Regardless of one’s opinion on this matter, there are certainly things to be learned from biological diversity, including a better understanding of conserved regulatory elements that may bridge diverse mitotic mechanisms.

As an example of informative mitotic diversity, one can look at mitotic behavior of the nuclear envelope. During mitosis in higher plants and animals the nuclear envelope disperses, allowing commingling of the nucleo- and cytoplasms. In many unicellular organisms, on the other hand, the envelope remains intact throughout mitosis. This appears to be a sharp distinction and a fundamental difference, but it turns out that there are examples of intermediate conditions that argue otherwise. In the green alga, *Chlamydomonas*, the nuclear envelope is largely intact, but near the spindle poles there is a window that allows centrosomes in the cytoplasm to extend MTs into the nucleoplasm and thereby affect chromosome position [75]. A similar situation is found in the nuclei of *Drosophila* embryos at the syncytial blastoderm stage [76]. In addition, there are cases where the spindle forms in the cytoplasm and the nuclear envelope remains intact. The mitotic chromosomes associate with the inner surface of the nuclear envelope and somehow form MT attachment sites on the outer surface of the envelope. As a result, the cytoplasmic spindle can organize chromosome segregation, even while the spindle MTs are excluded from the nucleoplasm [63,77,78]. These examples are all consistent with the simple view that mitosis requires a bi-polar array of MTs that interacts with the already duplicated chromosomes, linking sister kinetochores to sister poles. Exactly how this arrangement is achieved and regulated can vary, and may be of secondary importance.

With the advent of molecular biology, both to help identify the protein products of genes and to go from biochemical analyses to a set of genetic loci, mitosis research focused down on a relatively few organisms whose combination of genetic manipulability and susceptibility to molecular transformation and microscopic imaging made them useful experimental systems. Thus, as will become apparent in the chapters that follow, there is now a huge amount of information about the spindles of budding and fission yeasts, of the nematode, *Caenorhabditis elegans*, of the fly *Drosophila melanogaster*, and of mammalian cells grown in culture. Likewise, a few plant systems are under intense scrutiny. Yet, as the sequencing of DNA has become relatively inexpensive, and as molecular tools and capabilities have expanded, there is now a trend away from this biological myopia. We can look forward to a greater interrogation of biological diversity and a resulting improvement in understanding of this complex and important biological process. 

## 5. Biochemical Work to Characterize the Mitotic Machinery

The first successful efforts to purify mitotic spindles took advantage of the eggs of marine invertebrates, which could be obtained in large numbers and induced to enter an almost synchronous mitosis by the addition of the corresponding sperm [79,80]. Cell numbers, and therefore the amounts of isolated spindles were sufficient for biochemical study, but the first spindles isolated were non-physiologically stable and inert with respect to mitotic activity. Robert Kane improved this isolation protocol in 1962, leading to some characterization of the factors important for spindle stability [81]: pH around 6.0 and a not well understood aspect of lowering the solution’s dielectric constant. However, these spindles too were inactive, and even when the method was further improved by using glycerol and dimethylsulfoxide to preserve spindle stability in a reversible way [82], the isolates were still too inactive and complex to be very informative. The latter spindles showed some of the dynamic properties of spindles in living cells, e.g., sensitivity to hydrostatic pressure, but they did not move chromosomes, so it was hard to assess the functional significance of either their dynamic properties or any specific components. Still other media [83] were used to isolate labile spindles that yielded information about a few proteins that associated with spindle MTs in a polymerization-dependent way [84]. The discovery of Taxol as an agent to promote MT stability [85] spawned additional efforts to isolate useful mitotic spindles [86], but again, the lack of functional assays for the many components of the isolates frustrated attempts to use these preparations for a molecular analysis of mitotic mechanism. 

One of the most significant advances in spindle chemistry came from the lab of Edwin Taylor. It had been known for years that colchicine disrupted mitotic spindle structure and function but had little effect on a cell’s progression through interphase. Based on this specificity, Taylor surmised that the drug bound to the protein subunit of spindle fibers, i.e., the subunits of MTs. He made a radioactive form of this spindle poison and used then-standard methods of protein biochemistry to purify the colchicine-binding component of spindles [87,88]. Ironically, the material that provided the best yield of colchicine-binding protein was mammalian brain, a tissue known for its dearth of cell divisions. This observation provided important evidence that the colchicine-binding protein was used in multiple cellular settings, just as MTs had been found to be essentially ubiquitous. Subsequent work by Richard Weisenberg, identified conditions in which the isolated protein, now called “tubulin”, would assemble into MTs in vitro [89]. These advances opened up multiple approaches to the experimental study of mitosis.

Several labs used version of the conditions discovered by Weisenberg to make cell-free models of spindles that preserved aspects of spindle function and allow limited biochemical study, if not the analysis of components. This approach was pioneered by Hoffmann-Berling in the 1950s; he used glycerol to preserve aspects of the cytoskeleton as the cell membrane dissolved, then Mg^2+^-ATP to activate cellular contractility. He applied the same approach to mitotic cells [90], but with only limited success, perhaps because the conditions that preserved microfilaments and their interactions with myosin did not work for spindles. Maintaining a labile spindle became practical as soon as one knew methods to purify tubulin and promote its polymerization [89]. By lysing cells with non-ionic detergents in an equilibrium mixture of tubulin and MTs, investigators were able to support anaphase motions after membrane permeability was disrupted [91]. However, without a way to initiate anaphase in these lysed cells, one could only look for conditions that supported chromosome motion after it had started. For this purpose, solutions containing Mg-ATP and a high molecular weight polyethylene glycol were sufficient; soluble tubulin was unnecessary for anaphase A and limited anaphase B. Subsequent work showed that the presence of tubulin could increase the extent of spindle elongation [92], but mechanisms still remained elusive.

An alternative biochemical approach emerged from work on extracts of frog eggs. These huge cells can be broken and fractionated to yield undiluted meiotic cytoplasm in sufficient quantities to facilitate experimentation on factors that induce a mitotic state [93]. It was this system that allowed the first purification of “maturation promoting factor” [94], which later came to be known as cyclin-dependent kinase 1. This discovery attracted legions of workers to the frog egg system for biochemical analysis of cell cycle control. Upon the addition of frog sperm, this system will produce cycling nuclei and mitotic spindles whose action in a cell-free environment is remarkably life-like. These spindles have served as fruitful experimental material for many laboratories, as will be described in later chapters of this book. 

Another productive approach to spindle biochemistry was based on the use of labeled antibodies, which could identify and localize specific spindle components. Some investigators raised antibodies to proteins of interest, such as MT-associated proteins (MAPs) or motor enzymes and looked by indirect immunofluorescence to see whether these molecules were present in spindles [95,96,97]. This approach did, however, lead the field on some false trails, because the spindle contains a very large number of proteins, not all of which are functionally significant for mitosis. For example, the muscle protein, actin, appears to be a major spindle component [97], but later work showed that most spindle actin is monomeric; fibrous actin, which is more likely to be of mechanical importance, lies largely outside the spindle [98]. Nonetheless, the discovery through immuno-fluorescence that gamma-tubulin is localized at spindle poles [99] and that the minus end-directed motor, dynein, is localized to mitotic kinetochores [100,101] are examples of discoveries that opened a wealth of opportunities for experimentation, many of which will be discussed in other chapters. In summary, however, this rather targeted approach to component identification did not have the power necessary to reveal important but previously unknown spindle parts.

Substantial progress in the identification of spindle components was made serendipitously through the discovery that certain auto-immune syndromes in humans led to the production of antibodies that bound to unknown spindle components localized at intriguing sites, such as kinetochores [102]. The potential value of these antisera was recognized by several students of spindle structure, but it was the chromatin structure group at Johns Hopkins, led by Bill Earnshaw and Don Cleveland, that first capitalized on these sera as tools to identify centromere and kinetochore proteins [103]. This work produced a series of important papers in which centromere proteins (CENPs A–F) were identified as specific polypeptides. The significance of some of these proteins for mitosis was implied by the failure of mitosis following injection of these antibodies into mitotic cells [104]. CENP-A was purified and identified as a histone-like protein by the clever use of spermatozoa as a cell type that retains this protein, even as histones are removed to permit chromatin transport in a compact sperm head [105]. CENP-A and other kinetochore components identified in this way have become central players in mitotic function, as will be discussed in later chapters. 

## 6. Spindle Genetics as a Route to Understanding Mitotic Mechanism

Students of mitosis have long hoped that genetics would provide deep insights into the functionally significant components of the mitotic spindle. Hints supporting this hope came occasionally from the communities that studied organisms suitable for classical genetics, e.g., fruit flies. Mutants with interesting mitotic phenotypes were occasionally reported, e.g., some alleles of the *claret* locus, which is important for *Drosophila* eye color. These mutants included the phenotypes of abnormal mitoses in early embryonic cells [106] and of meiotic chromosome loss [107]. The reason for the surprising coupling of eye color with mitosis remained obscure for many years. It was only with the cloning of both the DNA around the *claret* locus and the gene for *Drosophila* kinesin that it became clear that this claret allele included a deletion of DNA from an adjacent gene, which encoded a motor enzyme important for mitosis [108].

Some workers sought mitotic mutants in normally diploid organisms by taking advantage of their biology. Ostergren used radiation mutagenesis of lilies and a screen for cells with aberrant mitosis during the brief haplophase that follows meiosis, but although he collected many such strains, the molecular tools need to analyze them were not available, so little progress resulted [109]. Likewise, students of fly biology sought male sterile meiotic mutants, maternal effect embryonic lethals, and late larval lethals in an effort to identify mutations affecting chromosome behavior and cell division. These mutants identified genes whose wild-type functions are important for chromosome condensation and integrity, for the progression of nuclear and centrosomal cycles, and completion of chromosome segregation. [110,111], but they did not cast light on mitotic mechanism. With only a genetic locus and a phenotype as guides, it was impossible to see mechanistic connections between mutant and wild type mitosis. 

A full scale genetic assault on mitosis, and on cell cycle regulation more generally, did not occur until Hartwell began his screen for temperature sensitive (ts) mutants of the budding yeast, *Saccharomyces cerevisiae* [112] and Nurse pursued the same strategy with the fission yeast, *Schizosaccharomyces pombe* [113]. Meanwhile, Morris undertook an analogous screen for ts mutants in *Aspergillus* with a specific focus on those with an aberrant mitosis [114], and Yanagida sought mutants of *S. pombe* that would pertain specifically to mitosis. His lab achieved singular success through the study of strains that drove a septum inappropriately through an undivided nucleus, the “cut” mutants [115]. Other students of yeast biology looked for mutations that led to an increased frequency of chromosome loss. These “chromosome instability” (CIN) mutants again identified genes important for high fidelity mitosis [116]. With the advent of DNA cloning for fungal genes by reversion of mutants to the wild phenotype through transformation with a library of wild type DNA, it became possible to learn the DNA sequences of genes whose mutation had led to mitotic failure. In this way both Morris’s lab and those of Rose and Hoyt discovered “pioneer” sequences of genes important for successful chromosome behavior in *A. nidulans* and *S. cerevisiae*. The mechanistic significance of several of these mitotic mutants became evident upon the cloning and sequencing of the gene for kinesin from *Drosophila melanogaster* [117]. Numerous fungal mutants with mitotic phenotypes could now be recognized as mutations in a family of MT-dependent motors that played important roles in the formation and function of mitotic spindles and related processes, such as karyogamy (the coming together of gamete nuclei to form a diploid nucleus). These genes and their analysis will be described in later chapters of this book. 

The ability to clone mutant genes in fungi with comparative ease led to an amazing spate of progress in identifying key molecular functions important for mitosis. Kinases that regulate the activity of both centrosomes and kinetochores were found in each of the organisms studied. Genes whose products were essential for chromosome attachment to the spindle and for centrosome duplication were discovered and analyzed. Among the most important discoveries made through a genetic approach was the identification of proteins that provide key functions in the spindle’s system of quality control, the “spindle assembly checkpoint”. As discussed in Chapter 5, clever genetic screens identified several proteins that contribute to a system that delays anaphase onset until the chromosomes are properly attached to the spindle [118,119]. Additional progress was made through a marriage of biochemistry with molecular and genetic approaches, as in the work by the Kilmartin, lab, who workers isolated both spindles and the “spindle pole bodies” (SPBs), which are the centrosomes of budding yeast, followed by a combination of raising antibodies and screening libraries to identify genes and gene products that were components of these important mitotic structures [120]. With the more recent rise of genome sequencing and mass spectroscopy researchers in mitosis have come to possess what is probably a complete “parts list” for the mitotic machinery of small cells. Again, this work will be reviewed in later chapters. In sum, the emergence of modern molecular technology has made a dramatic difference to the information about mitosis that can be gleaned from genetics.

## 7. Insights into Mitotic Mechanism from Studies of Mitotic Physiology In Vivo

Early cytologists described the structures and organelles involved in spindle assembly and function based on correlations between fixed and living cells. As they achieved greater clarity in the descriptions of mitotic events, they realized that an understanding of underlying mechanisms would require experimentation. Their investigations were supported by the development of companion technologies such as micromanipulation, microinjection, and fluorescent labelling of spindle molecules, all employed with a new mindset of reaching into the cell to perturb the mitotic process.

**a.**  **Experiments on Kinetochores**. Improved microscopy enabled descriptions of both localized and diffuse kinetochores, as seen in certain insects [121]. Experimental work using X-rays to fragment chromosome [122,123] provided direct evidence for the role of kinetochores in spindle attachment and chromosome segregation. X-ray induced fragmentation of chromosomes with localized kinetochore produced multiple fragments, only one of which contained a kinetochore. This fragments attached to the spindle and moved normally, but the fragments that lacked a kinetochore failed to attach and were lost at subsequent divisions [124]. By contrast, chromosomes with diffuse kinetochores were connected to spindle fibers by the entire poleward surface of each chromosome. X-ray-induced fragments of these chromosomes retained kinetic capacity; regardless of how small the pieces became, each chromosome fragment was pulled to opposite spindle poles by its associated kinetochore fibers [123]. This work established the importance of kinetochores in chromosome motion.
**b.**  **Observations and Experiments on Chromosome Movements.** Some diversity in anaphase was discovered by quantitative descriptions of chromosome movements in living cells. Changes in spindle length and kinetochore separation revealed two phases of chromosome movement [25,125]. One involved the shortening of chromosomal fibers, the other an elongation of the spindle with consequent chromosome movement. Early descriptive work revealed variation in the extent to which organisms relied on Anaphase A or B. Some used only Anaphase A (Tradescantia) and some only Anaphase B (Primary spermatocytes of the Aphid, Tamalia [125]). Some cells used both though separated in time (Secondary spermatocytes and embryonic cells of the Aphid, Tamalia; Hemiptera and Homoptera; [125]), and others used both anaphase mechanisms overlapping in time and therefore difficult to distinguish (grasshoppers; chick tissue culture cells [25]).

The two phases of anaphase were experimentally distinguished by their responses to chemical inhibition; chloral hydrate blocked anaphase B but not A [125]. In a different approach, Brinkley and colleagues [126] noted that low concentrations of colcemid had little effect on kinetochore fibers, but non-kinetochore spindle fibers were lost, resulting in a monopolar spindle. Other investigators exposed cells to hypertonic media, X-rays, or elevated temperatures and studied the abnormal anaphase configurations that arose as a result of cross-linked chromosomes (e.g., [127,128]). Following these perturbations, the forces that would normally have pulled chromosomes to the poles now pulled the spindle poles together, suggesting that chromosome-to-pole forces were stronger than the forces driving spindle elongation. The failure of these spindles to elongate correlated with a delay or absence of cleavage furrow formation, providing some of the first evidence that furrow formation is linked to the spindle. These and similar observations promoted the view that spindles comprised two components: the chromosomal fibers that pulled chromosomes poleward, and the spindle body that could elongate, pushing the spindle poles apart, indirectly moving the pole-attached chromosomes.

Early studies of spindle dynamics using polarized light were paralleled by detailed studies of chromosome behaviors in living cells using improved phase contrast optics and later, differential interference microscopy. The major features of chromosome movements during cell division were distilled from observations of plant endosperm divisions (Reviewed in [59]), as well as spermatocyte divisions in crane fly [128], grasshoppers [129,130], mantids [131], and phasmids [132]. In general, chromosomes moved along the curvature of the spindle, parallel to the spindle long axis. These movements are irregular, with occasional pauses and reversals of direction, but they are always directed towards one or the other spindle poles, and are always progressively becoming more centered on the equator of the spindle at metaphase. At anaphase, chromosomes separate, marking the end of metaphase, and move poleward, continuing on paths parallel to the interpolar spindle. These motions are independent but are far more coordinated than prometaphase motions. In both prometaphase and anaphase, localized kinetochores were seen to direct the independent movements of chromosomes.

**Figure 18:**
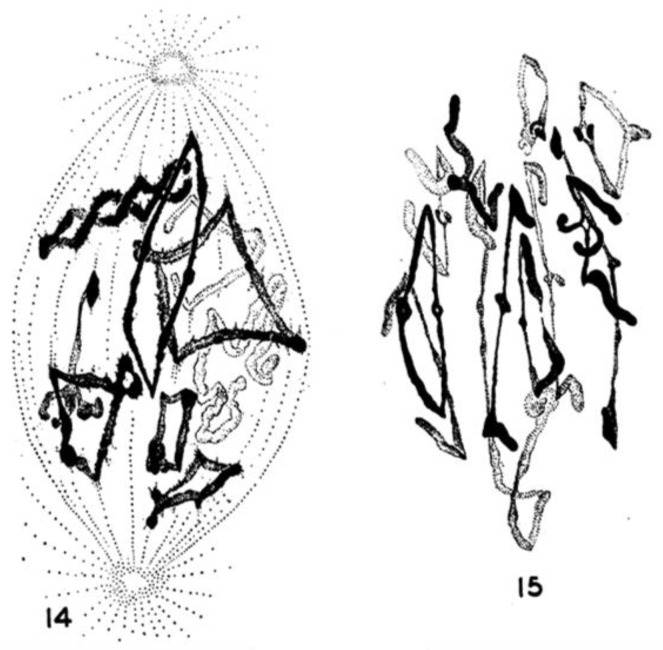
Typical pre-metaphase stretch of bivalent chromosomes in *Stagmomantis carolina,* a mantid. The kinetochores of homologous chromosomes are pulled far apart during Meiosis I in this species. 14 = early prometaphase, 15 is later. From Hughes-Schrader, 1943 [131]. With permission from the University of Chicago Press.

**c.**  **Chromosome Pulling Forces Were Detected in Prometaphase**. Descriptions of chromosome behavior in a variety of meiotic cells revealed a “pre-metaphase stretch” in which the distance between kinetochores on homologous chromosomes was greatly extended by action of the spindle [131,132], e.g., mantids and phasmids (Figure 18). This pre-metaphase stretch was followed by the gradual re-contraction of chromosomes and a resumption of normal prometaphase congression. Notably, the timing of the stretch and the resumption of congression were asynchronous on adjacent chromosomes, implying that mitotic forces acted independently on individual chromosomes. Taken together, these studies suggested the autonomy of chromosome movements during mitosis, eliminating models of collective transport by the spindle. These observations contributed to the rise of traction fiber models, in which kinetochores and their associated chromosomes were pulled individually towards the spindle poles to which they were attached.
**d.**  **Physical Perturbations of the Spindle as a Whole**. Some of the most convincing evidence for the existence of chromosomal fibers in vivo came from experimental manipulations of dividing cells. The centrifugation of mitotic cells distorted the spindle [121], stretching it [133] and/or severing chromosome attachment sites [134]. These perturbations also separated anaphase spindles into two half spindles, showing that chromosome-spindle pole attachments were strong enough to resist the centrifugal forces that distorted bivalent chromosomes. Centrifugation experiments further suggested a gel-like mechanical texture of the spindle.

The nature of this gel and of chromosomal fibers was further studied by the reversible application of hydrostatic pressure [49]. Pease found that progressive increases in hydrostatic pressure (e.g., 2000 to 6000 psi) progressively reduced the rate of anaphase chromosome movement, culminating in its complete cessation. Indeed, Pease provided early evidence for a functional linkage between the presence of a spindle gel structure and chromosome movement. When the rigidity of the fibrous spindle was reduced at elevated hydrostatic pressures, chromosome movement ceases. Remarkably, however, after a release of hydrostatic pressure, spindle fibers reassembled and chromosome movement restarted. Pease proposed that forces are imparted to the chromosomes by two phase transitions: a sol to gel transition, which added spindle fiber material and a solation of chromosomal fibers, which occurred at the spindle poles.

Quantification of spindle birefringence under various physical and pharmacological conditions provided key insights into the reversible assembly of the mitotic spindle (reviewed in [135,136,137]). A sharp dependency of spindle birefringence on temperature suggested a low energy bonding between aligned subunits of the birefringent spindle fibers and provided compelling evidence for the self-assembly of fibers at physiological temperatures [135]. Inoue and Sato [135] proposed the dynamic equilibrium model of spindle assembly in which aligned subunits forming birefringent spindle fibers were in equilibrium with a pool of unaligned, non-birefringent subunits. Consistent with Inoue’s equilibrium model, Edwin Taylor [138,139] showed that the protein synthesis required for spindle assembly is completed well before the onset of prophase. However, neither an appreciable loss of kinetochore fiber birefringence nor the predicted transient acceleration of poleward chromosome velocity in anaphase was observed upon the rapid cooling of crane fly or grasshopper spermatocytes [140].

In eggs of the marine worm, *Chaetopterus*, the metaphase-arrested spindle is attached to the egg cortex. When spindle MTs were depolymerized by exposure to cooling, colchicine or high hydrostatic pressure, the spindle shortened, transmitting forces along the chromosomal fibers to pull the metaphase chromosomes closer to the cortex [141]. Moreover, reversal of treatment, which allowed the repolymerization of spindle fibers, pushed chromosomes away from the cortex. Taken together these studies provided the first physiochemical evidence for a link between the reversible assembly of spindle fibers and force production for chromosome movements. The dynamic equilibrium model was later revised, based on the understanding that spindle birefringence is due largely to MTs and that spindle MTs self-assemble from a pool of tubulin subunits [142,143].

**Figure 19:**
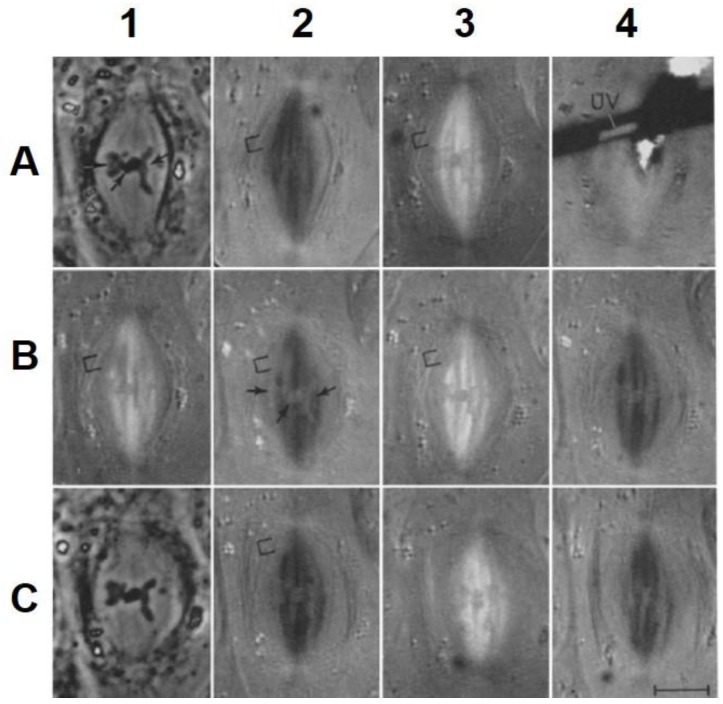
A crane fly spermatocyte irradiated during metaphase. (**A1**) autosomes labeled with arrow; (**A2**,**A3**) the position to be irradiated is indicated by a bracket; (**A4**) UV = the ultraviolet irradiation; (**B1**,**B2**,**B3**,**C2**) The position of the area of reduced birefringence (on the chromosomal fiber of the left bivalent) is indicated by a bracket; (**B2**) the autosomes labeled with arrows. The times of the photographs in minutes relative to the time of irradiation. A1, −11; A2, −7; A3, −5; A4, −0.5; B1, +2; B2, +2.5; B3, +6; B4, +7; C1, +10; C2, +14.5; C3, +18.5; C4, +19.5. The area of reduced birefringence moved to the pole, and did not displace the pole when it arrived there. From Forer, 1966 [144]. This image is displayed under the terms of a Creative Commons License (Attribution-Noncommercial-Share Alike 3.0 Unported license, as described at http://creativecommons.org/licenses/by-nc-sa/3.0/.

**e.**  **Local Perturbations of Spindle Structure and Function**. Experiments with microbeams of ultraviolet (UV) light raised provocative questions about the nature of MT dynamics and the roles of MTs in chromosome movements. Forer [144] reported that UV microbeam irradiation of chromosomal fibers in crane fly spermatocytes at metaphase produced localized “areas of reduced birefringence” (ARBs). ARBs subsequently moved poleward, even while the chromosomes remained aligned at the metaphase plate. Such observations indicated that chromosomal fibers were not static and suggested a continuous poleward flux of materials within the chromosomal fiber (Figure 19). Moreover, in anaphase, Forer reported variability in the impact of ARBs on chromosome movement [144]. Two thirds of the time, chromosome to pole movements ceased when irradiated fibers contained ARBs. One third of the ARBs, however, had no impact on chromosome movement. Moreover, two thirds of irradiated fibers did not develop ARBs, yet chromosome to pole movements were blocked as frequently as when an ARB formed. Forer interpreted these results to mean that chromosomal fibers contained two components: birefringent MTs, which neither produce nor transmit the forces required for chromosome movements, and a non-birefringent element that was required for traction the forces that pulled chromosomes poleward in anaphase A. Electron microscopy later confirmed that in at least some experiments, MTs within the ARBs were severed and/or depolymerized [145,146]. Forer’s complicated results from anaphase spindles have never really been explained.

With regard to spindle MTs that did not attach to kinetochores, a subset of these MTs from opposing poles were found to interdigitate at the spindle midzone (see above); these fibers exhibit even greater stability than was found with the fibers connected to chromosomes [147]. Experiments showed that this interpolar bundle was under compression from the forces pulling chromosomes poleward; when the bundle was severed by a UV microbeam, the spindle of a diatom collapsed [145]. Much progress has recently been made in understanding the organization and force production associated with the zone of overlap at the spindle equator, as discussed by Scholey et al., this volume [148].

**Figure 20:**
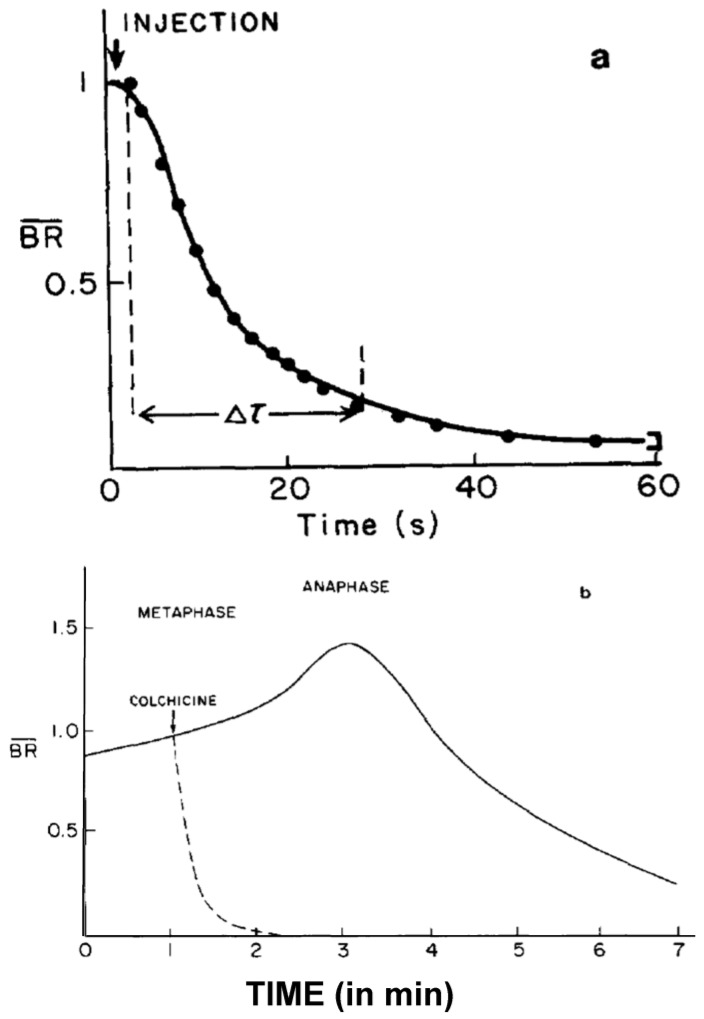
Changes in spindle birefringence (BR) in the half spindle as measured by a video spot meter. (**a**) Tracing of the chart record of the video voltage following injection of colchicine (0.2 mM) into a first division metaphase cell. The characteristic time, Δτ, of nonkinetochore MT depolymerization is measured by the time between the onset of BR decay and the time where the video voltage decreases to 10% of the initial value. The line is a first-order decay curve for k = 0.092. Spindle BR is normalized by the initial value at the time of injection; (**b**) Comparison of the rate of disappearance of normalized spindle BR after 1.5 mM intracellular colchicine injection with the normal rate of half-spindle disassembly at late anaphase. From Salmon et al., 1984 [149]. This image is displayed under the terms of a Creative Commons License (Attribution-Noncommercial-Share Alike 3.0 Unported license, as described at http://creativecommons.org/licenses/by-nc-sa/3.0/.

**f.**  **Studies on MT Dynamics in Vivo**. Salmon and coworkers [149] microinjected high concentrations of colchicine into dividing sea urchin eggs, blocking spindle MT polymerization, and allowing the rate of depolymerization to be measured. A rapid decrease in birefringence (BR) reflected the disappearance of non-kinetochore MTs, while kinetochore MTs were again differentially stable (Figure 20). Surprisingly, the calculated rate of depolymerization (180–992 dimers per second) was significantly faster than predicted from the in vitro parameters of tubulin dynamics. It was, however, consistent with a significantly different, “dynamic instability” pathway concurrently proposed for MT behavior [150]. At about the same time, several investigators capitalized on photobleaching, as well as on the incorporation of injected tubulin and a specially labeled tubulin, using fully functional tubulin “analogues” to interrogate the pathways of tubulin assembly and disassembly during mitosis and the mechanistic basis of chromosome movements [151,152,153].

In particular, fluorescence recovery after photobleaching (FRAP) experiments in sea urchin spindles in vivo revealed two phases of fluorescence recovery: a fast phase, reflecting a quick turnover of tubulin within the labile polar MTs (t_1/2_ = 19 s), and a slow phase associated with the comparatively stable kinetochore MTs (t_1/2_ = 60–90 s [153]). Moreover, photobleached bar patterns positioned within the half spindle showed no evidence for directional, poleward movement during the rapid recovery phase, indicating that poleward flux of unbleached subunits incorporated at the dynamic MT plus ends was not the mechanism by which fluorescence was recovering [154,155]. Instead, the fast recovery was explained by the dynamic instability of polar MTs. 

**Figure 21:**
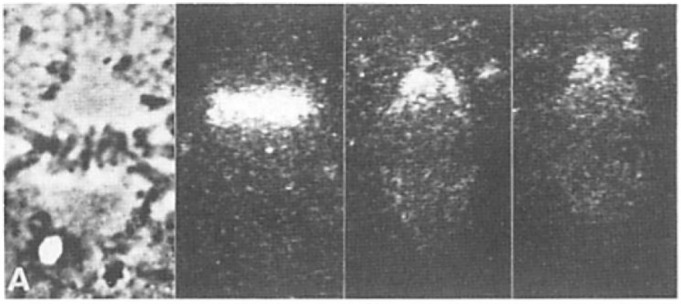
Phase-contrast and three fluorescence images of the cell to be analyzed. Times relative to photoactivation are (from left to right): 418, 5, 429, 674 s. Pole to pole distance = 18.8 µm. From Mitchison, 1989 [157]. This image is displayed under the terms of a Creative Commons License (Attribution-Noncommercial-Share Alike 3.0 Unported license, as described at http://creativecommons.org/licenses/by-nc-sa/3.0/.

In complete agreement with FRAP analyses, the incorporation of labeled tubulin injected into mammalian cells also show that non-kinetochore MTs quickly become fully labeled (<1 min) following the injection of fluorescent or biotinylated tubulin, while kinetochore MTs were not completely labeled a full 10 min after injection [156]. Further analysis of tubulin incorporation determined the sites of insertion, and removal, of tubulin subunits in the more stable kinetochore MTs, and identified a poleward flux of tubulin within kinetochore MTs [156,157]. Of particular note, Mitchison [157] developed a caged fluorescent tubulin that could be activated by a focused beam of UV light to turn on fluorescence in a narrow bar across mitotic spindles in vertebrate cells. Over time, the fluorescent bar moved toward the spindle pole, demonstrating a poleward flux of kinetochore MTs and their disassembly at spindle poles (Figure 21). These results added yet another layer of dynamics to kinetochore MT assembly with implications for the polar linkage of kinetochore MTs, as well as a potential mechanisms of force production that could power chromosome movement or the spindle elongation.

**g.**  **Investigating Spindle-Chromosome Interactions by Micro-Manipulation**. In a classic series of studies, Nicklas and coworkers used the tips of fine microneedles to tug on chromosomes in grasshopper spermatocytes and characterize their attachment to the meiotic spindle. They showed that chromosome-attached spindle fibers were essentially inextensible, that they were bound to the rest of the spindle near the pole, and that they were readily displaced, either laterally or toward the pole, without losing their chromosome attachment; moreover, each chromosome was quite independent of its neighbors [158]. Each chromosome behaved like a pendulum suspended by a thin wire and free to swing from a pivot point at the spindle pole. The chromosomal fiber was stiff under tension but flexible to compression and bending. Nicklas further demonstrated that during prometaphase and metaphase chromosomes could be detached from their fibers by repeated tugging with the needle. Once released from the needle, they would reattach to the spindle (See Supplementary Movie 1). In contrast, chromosomes in anaphase could not be detached from their fibers, despite great effort. The segregation of chromosomes following these micromanipulations was indistinguishable from that in control, unmanipulated cells.

Begg and Ellis [159] extended these observations, relating the mechanical connection between spindle and chromosome to the associated birefringent fiber. By selectively manipulating the chromosomal fiber they confirmed that this structure was attached to the chromosome. They showed that colchicine treatment abolished both the visible birefringent fiber and the mechanical connection between the chromosome and the spindle. During anaphase, if an already segregating chromosome was pushed poleward in advance of its neighbors, the birefringence of the associated kinetochore fiber was greatly reduced, presumably due to distortion of the fiber and the splaying of its MTs. Intriguingly, the splayed fiber remained intact and continued to shorten, as inferred from the eventual resumption of poleward movement by this chromosome in concert with its neighbors. These observations provided early evidence that the regulations of spindle-generated force and the mechanical linkage between spindle and chromosome changed at anaphase onset. During prometaphase, a kinetochore fiber that was not subjected to tension was unstable, and the linkage to the spindle could be lost as the chromosome reoriented. The onset of anaphase stabilized the chromosome’s spindle attachment, even though the chromosomal fiber was shortening. Indeed, the coupling between force production and mechanical linkage appeared to be fundamental to proper chromosome orientation and segregation [160,161].

Nicklas next interrogated the process of kinetochore orientation and reorientation during prometaphase [161]. Chromosomes were detached and monitored during reattachment. (For a visual description of this process, see Movie 1 in Supplementary Material. This video was made from an original film recording made by Nicklas and kindly donated for use in this review.) The results showed that kinetochore orientation was the primary determinant of the spindle pole to which that kinetochore would reattach. Regardless of where a detached chromosome was placed, the kinetochore interacted preferentially with the pole it most nearly faced. Second, once one kinetochore oriented towards a pole, the sister kinetochore was constrained to face the opposite spindle pole, as a consequence of chromosome structure. The bipolar configuration of each kinetochore favored the bipolar attachment of chromosomes.

Early in prometaphase, as chromosomes begin to engage with the spindle, kinetochore position and orientation are disorganized; almost any arrangement of kinetochore-MT interactions can be found (reviewed in [162]). Some of these initial arrangements result in inappropriate attachments between chromosomes and the spindle, e.g., sister kinetochores attached to a single pole. These attachments must be aborted and corrected for the spindle to accomplish proper chromosome segregation [158,160,161]. To study such reorientations, Nicklas and Koch [160] capitalized on their ability to induce chromosomal mal-orientations by micromanipulation. Chromosomes were detached, then bent and reoriented, so both of their kinetochores pointed towards and then attached to the same pole. This unipolar attachment resulted in an initial poleward movement of the mal-oriented chromosome, followed by a reorientation of one or the other kinetochore, so it faced the opposite pole, leading to a stable bipolar orientation (all visible in Movie 1). Reorientation of the manipulated chromosome was indistinguishable from the reorientations of spontaneously occurring mal-orientations. The manipulated chromosome, together with neighboring, unmanipulated chromosomes, entered anaphase and segregated normally during cell division. 

Since the spindle is applying tension to a chromosome when it is in a bipolar orientation, with sister kinetochores being pulled in opposite directions, Nicklas and Koch tested whether tension was the stabilizing factor in chromosome orientation. Tension was applied by using a microneedle to pull against the two kinetochore fibers of a mono-oriented chromosome, imitating the tension that would normally have been provided by each kinetochore’s attaching to the opposite spindle pole. Remarkably, applied tension induced stability in monopolar orientations, whereas control, monopolar chromosomes reoriented in the absence of tension. The importance of tension was further supported by experiments in which two chromosomes in monopolar orientations to opposite spindle poles where interlocked [163]. The poleward forces on one chromosome pulled against the poleward forces on the second chromosome, providing tension and stabilizing the interlocking configuration. In other work, Nicklas and coworkers, correlated the reorientation behavior of mal-oriented chromosomes with the distribution of kinetochore MTs [38,163,164,165]. Most MTs associated with a reorienting kinetochore appeared to have disconnected from the pole, though they were only slightly rotated from their initial orientations. Significantly, a few MTs did extend from the reorienting kinetochore towards the opposite pole. The origin of this minor population of MTs has not yet been determined. It is not known whether these MTs were captured from the opposite pole or initiated anew from the reorienting kinetochore.

This experimental work provided important evidence for a suggestion previously made by Dietz [128] that chromosome orientation and reorientation is based on selection; chromosomes explore multiple connections to the spindle, attaching and reattaching until a stable attachment is achieved. The difference between stable and unstable orientations is a result of the tension generated by forces acting to pull sister kinetochores toward opposite spindle poles. The stability of kinetochore linkage to the spindle is dependent on tension. The molecular mechanisms by which tension contributes to stability remains a key unsolved piece of the mitotic puzzle.

**h.**  **Assessment of Spindle-Generated Forces**. The first studies of mitotic force magnitudes considered the relationship between chromosome size and velocity. These studies showed that velocity was independent of size and thus of viscous load over a limited range. Chromosomes varying more than 2-fold in size exhibit the same speeds in both prometaphase and anaphase [130]. Similarly, McNeil and Berns [166] showed in metaphase PtK_2_ cells that when a single kinetochore is irradiated with a high powered laser, the unirradiated sister kinetochore transports twice the normal amount of chromatin at the same velocity as a normal anaphase chromosome.

Nicklas [130] and Taylor [167] estimated the force needed to move a chromosome at the speeds of mitosis through cytoplasm, whose viscosity they could estimate; both these calculations concluded that only ~1 × 10^−8^ dynes (0.1 pN) was required for anaphase motions in either grasshopper or newt cells. Subsequently, Nicklas measured the force an anaphase meiotic spindle could exert on a chromosome whose movement was impeded by a calibrated microneedle [168]. The force required to stop chromosome motion was 10,000 times greater than the force needed to overcome viscous drag. Forces less than ~10^−6^ dynes (10 pN) had no effect on chromosome speed; greater forces slowed the chromosome, with speed falling to zero as the opposing force approached ~7 × 10^−5^ dynes or 700 pN. Moreover, Nicklas estimated that the tension forces on a prometaphase chromosome (0.5 × 10^−5^ dynes; 50 pN) are significantly higher than the forces required for normal anaphase (10^−8^ dynes; 0.1 pN). The clear implication of these calculations and measurements was that spindles can generate far more force than is necessary simply to drag a chromosome through mitotic cytoplasm.

One significant implication of these large forces is the existence of a mechanism to regulate force production under normal conditions. Such a regulatory mechanism was also suggested by the load independence of chromosome velocity. Thus, regardless of how hard the mitotic motor can pull on a chromosome, the rate of chromosome movements is regulated, probably by kinetochore MT assembly/disassembly. Meanwhile, changing the length of kinetochore MTs can determine chromosome position [169,170]. Whether chromosome movements are powered by molecular motors or by MT dynamics remains under active investigation.

**i.**  **Experiments to Investigate Chromosome Congression to the Metaphase Plate**. Students of mitosis have long recognized the importance of metaphase in establishing a uniform initial condition for subsequent chromosome segregation. Numerous hypotheses were advanced for how chromosomes are brought to the spindle midplane. A simple and important idea emerged from the studies by Rashevsky [171], Hughes-Schrader [131], and Ostergren [172,173]: the poleward force on a chromosome might increase with distance from the pole. Paired chromosomes would then congress to the spindle equator because that position allowed the opposing forces to be balanced. Considerable evidence supports this force-balance theory, including observations on the consequences of upsetting the force balance. If sister chromatids are disconnected, either naturally at anaphase or artificially at metaphase [174], the opposing forces are uncoupled and each chromatid moves poleward. Similarly, a laser micro-beam can be used to destroy one metaphase kinetochore, and the chromosome then moves towards the pole to which the undamaged kinetochore is attached [166,175]. Thus, prometaphase and metaphase chromosomes are clearly being pulled in two directions at once.

One way to unify the fact of opposing tensions and Ostergren’s force-distance hypothesis is to suppose that the force applied to a kinetochore is proportional to the length of the kinetochore fiber. Evidence for this proposition was found in naturally occurring multivalent chromosomes, which were asymmetrically positioned on the metaphase spindle [176]. To test the force-length relationship more rigorously, the metaphase positions of experimentally generated multivalent chromosomes were analyzed in living grasshopper spermatocytes [177]. At metaphase, asymmetrically oriented, multivalent chromosomes lay closer to the pole to which the greater number of kinetochores attached. The quantification of the fiber bundle lengths for the grasshopper multivalents supported the hypothesis that the pole-directed force acting on a chromatid is linearly proportional to the kinetochore-to-pole fiber length. In a follow up study, Hays and Salmon [178] used a microbeam to ablate portions of a single kinetochore on metaphase bivalent grasshopper chromosomes. Irradiations of a single kinetochore caused the chromosome to shift to a new equilibrium metaphase position closer to the pole to which the unirradiated kinetochore was attached. After each experiment, the cells were fixed and examined by electron microscopy to determine the MT numbers on both the damage and the intact kinetochores. The greater the kinetochore damage, the fewer MTs remained, and the farther the chromosome position shifted. Assuming a balance of forces on the chromosome at metaphase, there was a direct correlation between the poleward force at the kinetochore and the number of kinetochore MTs. These results are consistent with models of chromosome congression in which the metaphase equilibrium position reflects a balance of forces. The results can be interpreted in light of “traction fiber” models in which poleward force producers are distributed along the length of kinetochore fiber MTs, or in the context of newer models in which kinetochore motors pull the chromosome poleward along the kinetochore MTs and against a “polar ejection force” that increases in strength towards the pole [179] (See also discussion below).

Polar-ejection, or “elimination” forces were originally discussed by Darlington [180] and Ostergren [173]. They have subsequently been characterized by the movements of mono-oriented chromosomes on monopolar spindles in which a single kinetochore fiber connects each chromosome to the pole. The sister kinetochore lacks a spindle fiber and is inactive. If the only mitotic forces on a chromosome were the poleward directed forces acting on kinetochores, then each chromosome should be pulled all the way to the pole. This is not observed. Rieder and colleagues [179] found that the positions of monopolar chromosomes and the oscillations they display are a result of both the pole-directed forces on kinetochores and forces associated with the polar array of MTs that push the chromosome arms away from the spindle pole. If the chromosomal arms are cut off by a laser microbeam, then the free arms are expelled radially from the polar region, while the smaller kinetochore fragment moves closer to the pole. Similarly, after cold or nocodozole treatment to depolymerize the polar MTs, the polar ejection forces are reduced and the mono-oriented chromosomes are pulled closer to the pole. 

The molecular basis of the polar ejection forces and their potential regulation in vivo is a subject of great current interest. Regardless of mechanism, the significant point is that the magnitude of force is maximal near the spindle poles and decreases with distance from the pole.

## 8. Models of Mitotic Mechanisms

The significant and intriguing events of mitosis have inspired many generations of scientists to speculate about the mechanisms that underlie chromosome segregation. At every stage in this segment of intellectual history, theoreticians have used the information available to propose ideas about the forces needed to cause chromosome motions. The earliest of these ideas were formulated before anyone knew the physical nature of proteins and nucleic acids, so the hypotheses put forward have in general not been helpful. For example, when looking at the spindles of marine eggs and embryos, physicists were impressed by their similarity to the lines of magnetic force visualized by iron filings or by probes of the electric field around a pair of fixed charges. Several models for mitosis were proposed, based on these similarities, but none of them led to experiments that clarified mitotic mechanism (reviewed in Wilson [15]). Other non-specific models based on colloids and exclusions between “phases” were similarly unproductive.

Cytologists who watched mitosis in the cells of diverse organisms recognized at least two sites of force generation: a “traction force” between the chromosomes and the poles to achieve anaphase A and an extensive force between the poles to produce anaphase B. Ideas about the origins of these forces depended on whether that scientist believed that the spindle fibers seen in fixed material were present in living cells. As Schrader pointed out in his essay “On the reality of spindle fibers” [121], one had to believe that the fibers in fixed cells represented either a structural feature that was cryptic in live material or lines of force produced by the spindle mechanism. The first ideas about mitotic mechanism that channeled thought in useful ways were ones that assigned contractile properties to the apparent connections between chromosomes and poles (summarized in [181]) and expansive properties to the interpolar fibers, first discussed by Bělař [127]. Actomyosin was commonly invoked for the former and an unknown fiber system for the latter. It was not until the quantitative study of spindle birefringence by polarization microscopy and the discovery of spindle MTs by electron microscopy that spindle modeling became specific.

Inoue and Sato [135] described a model for both chromosome to pole and pole-from-pole motions based on a labile association of the subunits that formed spindle fibers. They posited a dynamic equilibrium between soluble and assembled states of spindle subunits, using the formulation:

(1) $$A_{0}-B⇌B$$

where *A_0_* represented the total amount of polymerizable material and *B* the amount in polymer at any given time and temperature. A quantity proportional to *B* could be measured by polarization microscopy, allowing a reformulation of the equation simply in terms of the observable quantity, birefringent retardation (Г), where Г was assumed to be proportional to the amount of polymerized material, *B*. Thus, Equation (1) was rewritten

(2) $$\frac{{Г}_{0}}{k}-\frac{Г}{k}⇌\frac{Г}{k}$$

where k was the constant of proportionality. 

An equilibrium constant could then be computed directly from measured Г, since

(3) $$K_{eq}=\frac{Г}{{Г}_{\max}-Г}$$

By measuring Г as a function of temperature and assuming that Г_max_ was a good estimate of Г_0_, Inoue was able to estimate the Gibbs free energy, as well as ΔH and ΔS for the polymerization reaction. This analysis showed that spindle formation was entropy driven and allowed estimates of the force such a system could generate. However, this model for protein polymerization was hard to relate to then current ideas about polymer assembly [182]. A reformulation of the data in the context of normal polymerization reactions brought the initial interpretation into concurrence with ideas about protein polymerization and was important advance [141]. Such in vivo experiments are, however, subject to the concern that changing the temperature of a cell may affect processes other than spindle birefringence, such as the concentration of free calcium ions or various important nucleotides, as well as the properties of membranes. Nonetheless, the idea that MT assembly and disassembly might be the source of forces for chromosome movement gained wide-spread attention and favor, dominating the field for several years.

Alternative views were provided from several sources. Some scholars were staunch supporters of the idea that actin and myosin were likely to be the sources of all intracellular motions, mitosis included. Arthur Forer supported this view by experiments that implied a lack of correlation between spindle birefringence and the ability of spindles to exert forces on chromosomes [183]. He went on to find evidence for actin in spindles, a view supported by later work with immunofluorescence. Subirana developed an hypothesis for how spindle fibers, e.g., actin, might interact with matrix components through a mechanochemical enzyme, such as myosin, to push these fibers through cytoplasm in directions defined by their intrinsic polarity [184]. However, a substantial body of work with both actin inhibitors and probes for actin localization has largely discredited this point of view. Actin and related proteins may play roles at some sites of spindle force generation, such as some kinetochores during spindle fiber attachment [185]. Moreover, in very large spindles there is now compelling evidence that actin contributes to the gathering in of chromosomes during prometaphase, facilitating subsequent attachment of kinetochores to spindle fibers [186]. Other than that, most thinking about mitotic forces has focused on MTs.

Following the discovery of axonemal dynein [187], investigators realized that MT-dependent ATPases could be the major source of mitotic forces. One model explored the possibility that forces for chromosome motion were generated between spindle MTs by a dynein-like motor enzyme [48]. This proposal considered chromosomes simply as passengers attached to a subset of spindle MTs. The model was sufficiently specific that the authors found only one set of MT orientations that would allow a single motor to accomplish the then known functions of mitosis. While the idea was attractive and widely considered, the model’s assumptions about spindle MT polarity were subsequently shown to be wrong [56,57,188]. The idea of motor-driven interactions between MTs as a mechanism for anaphase B has persisted in a useful way, as discussed in Scholey et al., this volume [148], but as a way to achieve anaphase A and other aspects of chromosome motion, such as congression to the metaphase plate, this model must be dismissed.

With the discovery of kinesin and its role in vesicle motion, motor enzymes re-entered mitotic modeling with the postulate that kinetochores might be sites of motor localization, allowing chromosomes to move over spindle MTs in an active way. This important innovation was a departure from the longstanding idea that chromosomes were simply passengers for whatever mitotic force generators might exist. Mazia expressed this view most lucidly with the adage that “the role in mitosis of the chromosome arms, which carry most of the genetic material, may be compared with that of a corpse at a funeral: they provide the reason for the proceedings but do not take an active part in them” [189]. With advances in the genetics and molecular biology of yeasts and fruit flies, it became clear that cells contain a large repertoire of kinesins and dyneins, so there was no dearth of motors to help chromosomes move. As the kinesin super-family grew in size and as it was realized that even a simple yeast cell made five or more kinesins, as well as a cytoplasmic dynein, the ideal of a simple model for mitosis faded rapidly. Data on motor localization demonstrated that there were force generators at kinetochores, on chromosome arms and poles, on spindle fibers, in asters, and at the cell cortex. The question became one of learning how much each of these motors contributed to each kind of chromosome motion. These issues are discussed in later chapters of this book.

As the polymerization behavior of tubulin was elucidated by experiments on purified components in vitro, the idea of MT dynamics as an origin for mitotic forces resurfaced in a new and exciting form. One of the most innovative models was based on the fact that MTs at apparent polymerization equilibrium are actually at steady state; they can polymerize at one end and depolymerize at the other, making individual polymers into treadmills [190]. While this property might appear to violate the second law of thermodynamics, no such problem is encountered, because the system is driven by hydrolysis of the GTP bound to tubulin during its polymerization. This model envisioned kinetochores as sites of MT attachment that were able to allow tubulin addition during metaphase, so MTs could treadmill poleward, losing subunits at the spindle poles. The kinetochore was endowed with the capabilities of either allowing tubulin addition or not, so at anaphase onset it ceased to permit polymerization and then followed the MT plus end as it was pulled poleward by tubulin loss at the MT minus ends near the spindle poles. It is noteworthy that many complex aspects of MT dynamics that have recently been observed with markers for MT movements are completely consistent with the predictions of this model. 

The discovery of dynamic instability [150] added further interest and complexity to the properties of MT dynamics. One of the most important tasks of the mitotic spindle is to form stable attachments with kinetochores such that sister chromatids are linked to sister poles. The behavior of dynamically instable MTs led to an insightful model for the attachment process [191], which has subsequently been developed in quantitative form by several groups [192,193]. Recent work on this important issue will be discussed elsewhere in this book.

Theoreticians of the 1980s were also interested in MT dynamics as a potential force generator, so in spite of the lure of motor enzymes, several papers were published, developing this idea. One of these explored MT (or actin) dynamics as a source of mechanical work, using principles of thermodynamics [194]. Another analyzed a structural model for the attachment between spindle MTs and the kinetochore, based on a “sleeve”, as suggested earlier by Margolis and Wilson [190], that would surround the polymer end and provide a movable attachment between a kinetochore and MTs as the polymers shortened [195]. Shortly thereafter, the Kirschner group obtained evidence that depolymerizing MTs could retain their attachment to kinetochores in vitro [195]. These authors suggested that this retention was sufficiently strong to allow MT depolymerization to generate the forces necessary for anaphase A, either by a sleeve of the kind proposed by Hill or with a ring-like structure that could surrounded the MT and ride a “conformational wave” that might be generated by MT depolymerization [196]. 

These ideas received considerable support from subsequent observations, both in vitro and in vivo. Members of the McIntosh lab demonstrated minus end-directed chromosome motion in vitro; isolated chromosomes could follow the depolymerizing end of shortening MTs at physiological speeds in the absence of soluble nucleotide triphosphates [197]. This and subsequent work by this and other groups on the generation of force by MT depolymerization will be reviewed in a later chapter. It is noteworthy, however, that the observations cited above made the experimental landscape of spindle physiology remarkably complex. There is force generation by MT depolymerization, by motors of the kinesin super-family, and by dynein, all present and active in the spindle. Modeling chromosome motion is now a significant challenge. Future progress will require a painstakingly careful union of a large body of facts about each cell type under consideration and a deep knowledge of the relevant chemical physics.

Anaphase B, one the other hand, is enough simpler that several useful models for spindle elongation have been put forward. Early work, formulated in the context of the idea that those spindle MTs not connected to chromosomes were “continuous”, led to the straightforward proposal that spindles grew longer simply by the elongation of their component MTs [135]. Following the discovery that continuous MTs are rare, if they exist at all, and that the interpolar spindle is made from two interdigitating MTs families, one emanating from each pole [34,198], the concept of spindle elongation by motor-driven sliding of antiparallel MTs has been favored [199,200]. More recent and complete work on spindle elongation in fruit flies has greatly extended and formalized those ideas with a quantitative model that is a rigorous accounting of a considerable amount of structural, biochemical, and physiological data, as reviewed in Scholey et al., this volume [148].

Knowledge about small spindles, such as those that form in yeast cells, is now sufficiently complete that several groups are trying to formulate quantitative models with predictive power [201,202]. These models too will be addressed in subsequent chapters, but in summary we can say that our knowledge of mitotic phenomenology is now sufficiently complete that the dream of a single “model for mitosis”, cherished by many earlier investigators, is simply that: a dream. The biological significance of mitotic events is sufficient that cells have invoked multiple mechanisms to achieve the goal of accurate chromosome segregation. The book that follows is a testimony to the skill and hard work of a small army of investigators who have contributed to our current understanding of the process. While no simple model for mitosis is currently available, or perhaps ever will be, our thinking about the process is becoming ever more precise, providing important new understanding about key molecules and targets that may well advance our clinical treatments for human disease. We can plausibly hope that the mechanisms now understood will comprise the framework for a deep understanding of mitosis in the not too far distant future. 

## 9. Conclusions

This chapter describes a remarkable series of advances in our understanding of a complex and important biological problem: the mechanisms by which eukaryotic cells transmit a complete, undamaged genome to their daughters. The progress made reflects not only the skill, diligence, and cleverness of many investigators, it shows how each generation of scientists has depended on both previous progress in descriptions of natural phenomena and on the development of new technologies. The small size and delicate structure of mitotic spindles has made them a “poster child” for the importance of technological advances, first in microscopy, then in the intricate ways that genetics, biochemistry, and molecular biology can cooperate to identify important functional components of a complex process. In addition, however, the research on mitosis described here has demonstrated the importance of mechanical processes in cell organization and physiology. We hope that future generations of biologists will take note of this important aspect of cell biology.

## Supplementary Materials

The following are available online at www.mdpi.com/2079-7737/5/55/s1.
